# Remote monitoring of patients with rheumatoid arthritis in a low disease activity state: a mixed methods evaluation across six hospitals in London, UK

**DOI:** 10.1093/rheumatology/keae112

**Published:** 2024-02-24

**Authors:** Kathryn Watson, Helen Sheldon, Elena Pallari, Nikita Arumalla, Rachel R Olive, Olga Boiko, Camille Aznar, Emma-Jayne Adams, Ailsa Bosworth, Len Demetriou, Melanie Martin, Mary-Ann Palmer, Polly Sinclair, Emily J Smith, Nick Sevdalis, Andrew Walker, Toby Garrood

**Affiliations:** Centre for Implementation Science, Institute of Psychiatry, Psychology and Neuroscience, King’s College London, London, UK; Insights Team, Health Innovation Network, London, UK; Insights Team, Health Innovation Network, London, UK; Rheumatology Department, Guy’s and St Thomas’ NHS Foundation Trust, London, UK; Centre for Implementation Science, Institute of Psychiatry, Psychology and Neuroscience, King’s College London, London, UK; Centre for Implementation Science, Institute of Psychiatry, Psychology and Neuroscience, King’s College London, London, UK; Insights Team, Health Innovation Network, London, UK; Patient Author; National Rheumatoid Arthritis Society, Maidenhead, UK; Centre for Implementation Science, Institute of Psychiatry, Psychology and Neuroscience, King’s College London, London, UK; Rheumatology Department, Guy’s and St Thomas’ NHS Foundation Trust, London, UK; Patient Author from Guy’s and St Thomas’ NHS Foundation Trust, London, UK; Insights Team, Health Innovation Network, London, UK; Rheumatology Department, Guy’s and St Thomas’ NHS Foundation Trust, London, UK; Centre for Implementation Science, Institute of Psychiatry, Psychology and Neuroscience, King’s College London, London, UK; Insights Team, Health Innovation Network, London, UK; Rheumatology Department, Guy’s and St Thomas’ NHS Foundation Trust, London, UK

**Keywords:** remote monitoring, rheumatoid arthritis, service evaluation, implementation

## Abstract

**Objectives:**

This study evaluated the scale-up of a remote monitoring service, capturing monthly Rheumatoid Arthritis Impact of Disease scores and patient-generated text messages, for patients with rheumatoid arthritis (RA; in remission or with low disease activity) attending routine outpatient clinics across six hospitals. We explored patients and staff experiences and implementation outcomes.

**Methods:**

A pragmatic, mixed methods approach was used, with active patient involvement throughout. We undertook a rapid review, analysed service-level data, and conducted a patient survey and patient and staff interviews, informed by the Capability, Opportunity, Motivation, Behaviour (COM-B) and Exploration, Preparation, Implementation, Sustainment (EPIS) theoretical frameworks.

**Results:**

The review included 37 articles, covering themes of patient and clinician acceptability, engagement, feasibility and clinical impact. Service-level data (*n* = 202) showed high levels of patient engagement with the service. The patient survey (*n* = 155) showed patients felt the service was easy to use, had confidence in it and felt it improved access to care. Patient interview (*n* = 22) findings mirrored those of the survey. Motivating factors included increased responsiveness and ease of contact with clinical teams. Views from staff interviews (*n* = 16) were more mixed. Some implementation barriers were specific to roll-out sites. Prioritization of staff needs was emphasized.

**Conclusion:**

Patients were positive about the service and engagement was high. Staff views and engagement were more mixed. Results suggest that equal levels of patient and staff engagement are required for sustainability. These findings further our understanding of the implementation challenges to scaling remote monitoring interventions for patients with rheumatoid arthritis in routine care settings.

Rheumatology key messages

- Patients were overwhelmingly positive about the two-way remote monitoring service that captured 4-weekly RAID scores.
- Staff views were more mixed and engagement beyond the pilot site was low.
- Equal levels of patient and staff engagement are required for sustainability of the service.

## Introduction

Rheumatoid arthritis (RA) is a chronic inflammatory joint disease affecting around 1% of the UK population [1]. RA is a fluctuating condition marked by exacerbations, known as ‘flares’. Without appropriate treatment, progressive joint damage and irreversible disability can occur [2]. Modern treat-to-target approaches involve frequent monitoring of disease activity, with the goal of disease remission (or low disease activity) [2]. In the UK, National Institute for Health and Care Excellence (NICE) guidelines recommend patients should be involved in decisions about their care and have rapid access to specialist care for flares [3]. Similar guidelines exist in other countries, and the European Alliance of Associations for Rheumatology (EULAR) has recently published recommendations supporting the inclusion of self-management advice and resources in the routine management of people with RA [4].

Patient reported outcome measures (PROMs), when used effectively, can support patient-centred care [5]. One example is the Rheumatoid Arthritis Impact of Disease (RAID) score, a multidimensional, validated PROM covering seven domains (pain, functional disability, fatigue, sleep, coping, physical and emotional well-being), which has been found to discriminate between active and non-active disease [6]. The utility of RAID in routine care settings and its strength in identifying patients with unmet needs has also been demonstrated [7]. RAID produces ratings that correlate with the 28-joint Disease Activity Score (DAS28), a composite score that captures and characterizes disease activity and is used to determine treatment thresholds in clinical practice [7, 8].

Incorporating PROMs, such as RAID, into online systems offers potential benefits [5]. The Remote Monitoring of RA (REMORA) study designed and tested a smartphone application (app) that enabled people with RA to monitor and report daily symptoms, with data integrated into their electronic health record (EHR) and summarized graphically to inform clinical consultations [9]. The study demonstrated acceptability and feasibility of REMORA. A similar mobile app called RAConnect has demonstrated high usability in initial evaluation [10]. Functions included collection of patient-reported data, patient-generated free texts and generation of email reports. Other studies that have shown promising results include a randomized controlled trial of a smartphone app [11], and a cloud-based platform to provide improved holistic care for patients with RA, consisting of aggregated clinical and home monitoring data [12].

Due to the unpredictable nature of RA and often arbitrary appointment scheduling necessitated by capacity constraints, effective care planning can be challenging. Embedding electronic PROMs into clinical practice within outpatient settings could add substantial value. For example, collecting PROM data outside of clinic visits could lead to early identification of disease flares and more efficient appointment allocation, ultimately leading to time and cost savings for patients and the NHS, as well as better disease outcomes. The need to achieve such efficiencies has been widely recognized [13, 14], but was brought to the fore by COVID-19. It is therefore a priority to evaluate remote monitoring interventions that can promote patient-centred care and provide opportunities for improved clinical management in RA. In particular, we need to better understand the factors that influence implementation of remote monitoring technologies at scale and over extended periods of time within routine care, i.e. beyond research settings [8–10].

To address this need, we report an evaluation of the scale-up of a two-way remote monitoring service for patients with RA attending routine outpatient services across six southeast London National Health Service (NHS) hospitals. Specifically, we conducted a pragmatic, mixed method evaluation focused on exploring (i) patients and staff experiences, and (ii) implementation outcomes, including patient and staff acceptability, feasibility, and barriers to and drivers of implementation.

## Methods

### Description of the remote monitoring service

The remote monitoring service has been briefly described elsewhere, including the user-centred design approach [15–19]. Patients who met the eligibility criteria, namely those in remission or with low disease activity (Supplementary Data S1, available at *Rheumatology* online), were invited to join the service during routine consultations and were onboarded by a Digital Pathway Coordinator (DPC) (Supplementary Fig. S1, available at *Rheumatology* online).

After onboarding, patients were invited, via a link texted to their smartphone, to complete a RAID score every 4 weeks (Supplementary Fig. S2, available at *Rheumatology* online). An option to submit free text responses was presented following RAID completion, alongside graphical representation of RAID scores [19]. Automated reminders were sent at intervals and patients could opt out at any time.

Responses were monitored by the Digital Path Coordinator working closely with service teams. An automated flagging system highlighted elevated RAID scores or incoming text messages. The Digital Path Coordinator managed the flow of patient data to enable clinicians to make triage decisions about whether patients needed an appointment or remote advice (Supplementary Fig. S1, available at *Rheumatology* online).

**Figure 1.**
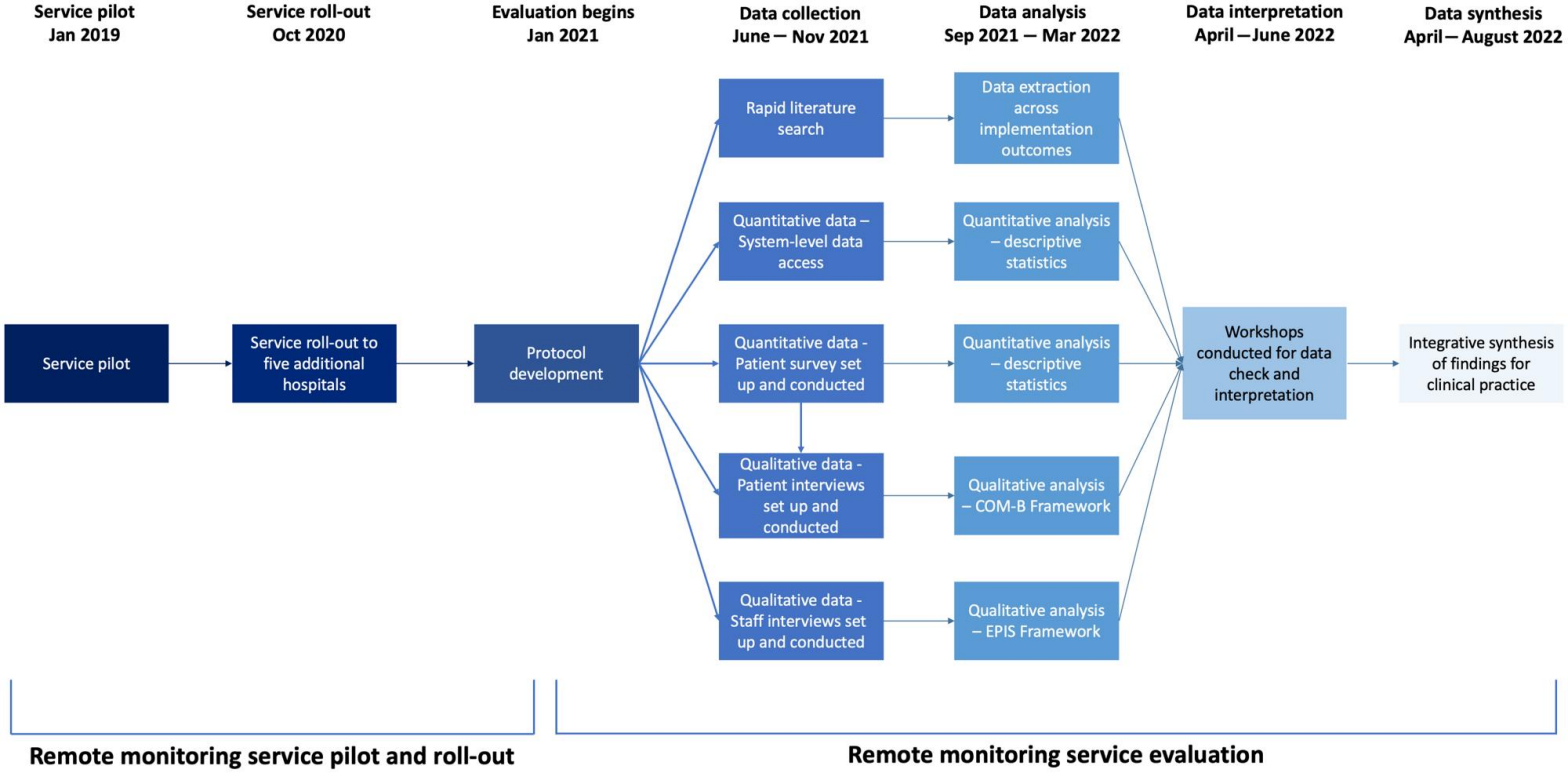
Timeline of the remote monitoring service pilot and evaluation. COM-B: Capability, Opportunity, Motivation, Behaviour framework; EPIS: Exploration, Preparation, Implementation, Sustainment framework

The service was piloted in 2019 with promising results [15]. In October 2020, the service was rolled out to five further hospitals. This evaluation took place from January 2021 to August 2022 (Fig. 1).

### Study design

The evaluation team consisted of a partnership between the Clinical Product Owner, Clinical Lead, DPC, clinicians, researchers, patient and public involvement (PPI) coordinators and patients.

**Figure 2.**
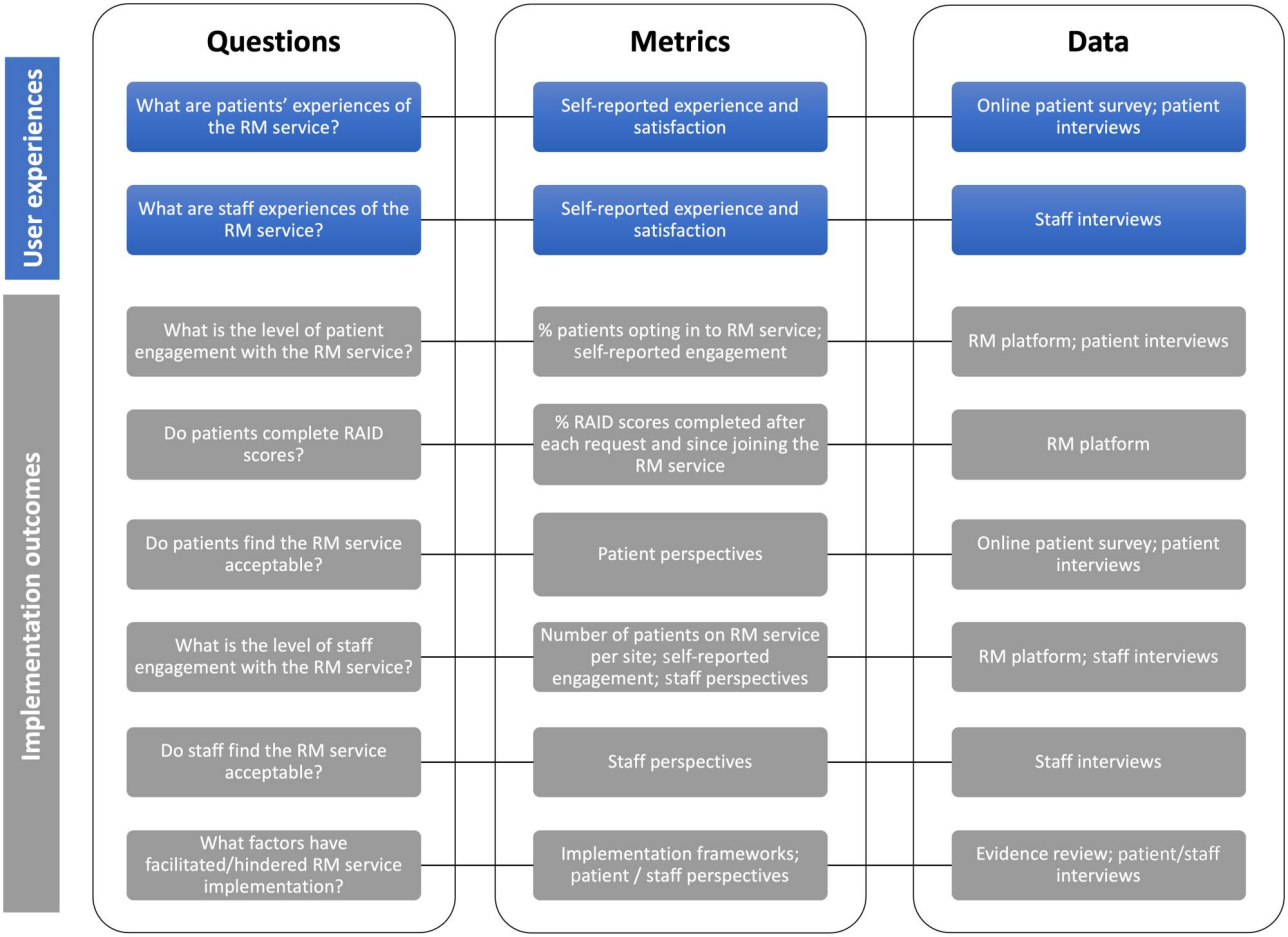
Framework for the remote monitoring service evaluation. RAID: Rheumatoid Arthritis Impact of Disease score

A pragmatic, mixed methods approach was used (Fig. 2), which consisted of a rapid evidence review, analysis of service-level data, patient survey, and interviews with patients and staff. Evaluation design, analysis and interpretation were discussed at workshops to facilitate patient feedback, refine key findings and address contradictory views. An early protocol was developed [20] and shared with stakeholders, including the project steering group, local commissioners and a wider group of patients.

### Rapid evidence review

The rapid evidence review involved database searches of Medline and Web of Science (September 2021), combining the following search/MESH terms: Rheumatoid Arthritis AND remote monitoring OR mobile app OR mobile applications OR smartphone app OR smartphone application OR telehealth OR tele-health OR telecare OR tele-care OR electronic health OR mhealth OR ehealth. Inclusion criteria were research papers and protocols published in English. Articles focusing on ‘telemedicine as virtual consults’ were excluded. Study identification and data extraction was conducted in Microsoft Excel (by O.B. and N.A.). Discrepancies were resolved via consensus discussion.

### Quantitative data—system-level data and online patient survey

Anonymized service-level data from January 2019 to October 2021 were extracted from the remote monitoring platform (by E.S.) and underwent quantitative analysis using Microsoft Excel (by K.W.). Analyses focused on evaluating patient engagement and included only data from patients meeting the eligibility criteria.

For pragmatic purposes, the patient survey was designed to largely replicate an earlier survey conducted at the pilot site. It consisted of 40, predominantly closed-response questions that covered acceptability, ease of use and demographics (Supplementary Data S2, available at *Rheumatology* online), and was set up on MS Forms (by H.S. and P.S.). Invitations were sent, via the remote monitoring service, to all 315 patients opted-in to the service in October/November 2021. In total, 163 completed responses were imported into SPSS Statistics (IBM Corp., Armonk, NY, USA). Analysis of the 155 responses from users of the service was conducted in SPSS Statistics and Microsoft Excel (by P.S. and K.W.). Descriptive statistics were used to summarize findings.

### Qualitative data—patient and staff interviews

Patients were recruited via the patient survey. Thirty-two patients responded to the invitation and 22 were interviewed. This included all those from the roll-out hospitals and a convenience sample from the pilot hospital. Purposive sampling was used to identify 26 staff members, of whom 16 were interviewed, working across different roles within rheumatology outpatient services at the six hospitals. Staff were invited via email.

Semi-structured interview guides were informed by two established implementation frameworks, Capability, Opportunity, Motivation, Behaviour (COM-B) [21] and Exploration, Preparation, Implementation, Sustainment (EPIS) [22], for patient and staff interviews, respectively. Patient interviews focused on overall experience of using the remote monitoring service, while staff interviews also explored implementation (Supplementary Data S3, available at *Rheumatology* online). Guides were refined after initial interviews. After gaining consent, interviews were conducted virtually or face-to-face. Audio recordings were transcribed professionally, identifiable information was removed, and coding was conducted in Microsoft Excel.

Transcripts were analysed using framework analysis [23] and deductive and inductive coding approaches (by H.S., E.P., R.O., C.A., K.W. and O.B.). Ten percent of patient interview data were double coded (by K.W. and O.B.) and consensus was reached on the thematic structure.

### Ethics

The study was reviewed and approved by Guy’s and St Thomas’ NHS Foundation Trust as a service evaluation. This type of study conducted in an NHS setting does not require NHS ethical approval. The study used anonymized routine monitoring data collected under GDPR article 6 1 (e) and article 9 2 (h). Written informed consent was obtained from participants for all other data (i.e. collected via interview and survey) prior to data collection.

## Results

### Rapid evidence review

The literature search yielded 44 articles of which 37 met the criteria for inclusion: 12 trials, 10 reviews, 10 qualitative/implementation outcome studies and 5 study protocols. Studies prioritized the patient perspective. Identified themes of relevance to implementability and scalability of remote monitoring services included patient and clinician acceptability, engagement, feasibility and clinical impact (Table 1 and Supplementary Table S1, available at *Rheumatology* online).

**Table 1.** Rapid evidence synthesis review summary

Study authors^a^	Year	Country	Patient acceptability^b^	Clinician acceptability	Patient engagement^c^	Clinician engagement^c^	Feasibility	Clinical impact	Available apps
Nishigushi *et al.*	2014	Japan	✓						
Azevedo *et al.*	2015	Portugal	✓						
Epis *et al.*	2016	Italy	✓						
Miedany *et al.*	2016	UK						✓	
Riel *et al.*	2016	Netherlands	✓						
Salaffi *et al.*	2016	Italy	✓					✓	
Yen *et al.*	2016	USA	✓						
Grainger *et al.*	2017	New Zealand							✓
Reade *et al.*	2017	UK			✓		✓		
Walker *et al.*	2017	Switzerland	✓	✓			✓		
Mollard and Michaud	2018	USA	✓		✓				
Gandrup and Yazdany	2019	USA	✓						
Luo *et al.*	2019	USA							✓
Najm *et al.*	2019	France	✓						✓
Navarro-Millan *et al.*	2019	USA	✓						
Austin *et al.*	2020	UK	✓	✓					
Bhattarai *et al.*	2020	Australia	✓						
Grainger *et al.*	2020	New Zealand	✓	✓					
Knitza *et al.*	2020	Germany	✓						
Krusche *et al.*	2020	Germany		✓		✓			
Kuusalo *et al.*	2020	Finland	✓					✓	
Mollard and Michaud	2020	USA			✓			✓	
Najm *et al.*	2020	France	✓		✓				
Seppen *et al.*	2020a	Netherlands			✓				
Seppen *et al.*	2020b	Netherlands						✓	
Sharp *et al.*	2020	UK	✓					✓	
Chahal *et al.*	2021	Canada and USA	✓						
Lambrecht *et al.*	2021	Germany	✓						
Lee *et al.*	2021	USA	✓	✓				✓	
MacIver *et al.*	2021	UK						✓	
Magnol *et al.*	2021	France			✓				
Muskens *et al.*	2021	Netherlands			✓			✓	
Nowell *et al.*	2021	USA	✓						
Richter *et al.*	2021	Germany	✓	✓					
Shaw *et al.*	2021	Switzerland	✓		✓				
Uhrenholt *et al.*	2021	Denmark	✓						
White *et al.*	2021	UK	✓						

a For references, see Supplementary Table S1, available at *Rheumatology* online.

b Issues relating to the usability of the intervention, and/or to patient knowledge, motivation and preferences.

c Issues relating to barriers and challenges in the implementation of the intervention.

Patients found remote monitoring interventions acceptable as a means of communication with clinicians and to allow review of their own symptoms [12, 24–28]. Remote monitoring interventions also provided support, for example, with medication issues and disease education [28]. However, patients expressed concerns about technical skills and that regular monitoring could potentially lead to harm, such as an over-focus on pain [27, 29, 30]. Clinicians also found remote monitoring interventions useful [9, 25] but expressed concern about patient and clinician technical abilities and worry that disease-related anxiety could be increased through quantifying self-reported measures [10].

Patients engaged with remote monitoring interventions successfully if the user interface was simple, visual and incorporated automated reminders [30]. Barriers to engagement included high satisfaction with usual management approaches, poor motivation due to lack of symptoms and the system acting as a disease reminder [27, 31]. Evidence regarding clinician engagement was more limited. Some studies reported lack of awareness of remote monitoring interventions; others found them time consuming and complicated [32].

Four studies found non-inferior RA disease activity or remission rates while using remote monitoring interventions [25, 31, 33, 34]. In contrast, two studies found a significant reduction in time to achieve Clinical Disease Activity Index (CDAI) remission scores and mean DAS28 scores [35, 36]. There was a reduction in numerical clinic visits used by the intervention group [36] alongside greater medication adherence [33], but one study reported increased nurse telephone contacts due to ePROM trigger thresholds, with similar levels of other healthcare use between groups [34].

### Quantitative analysis

#### Remote Monitoring system-level data

Data extraction identified 202 eligible patients. Patients from all hospitals were represented, but most were from the pilot hospital (154; 76%). This mirrored the pattern of clinician engagement across the sites. During the 34-month study period, 92% of patients remained onboard the service. The majority of RAID requests (83%, *n* = 2974) were completed, of which 50% were completed following the first request, and 72% within 4 days (Supplementary Fig. S3, available at *Rheumatology* online). Average RAID completion rates per patient remained consistently above 80% irrespective of time since joining the service (Supplementary Table S2, available at *Rheumatology* online).

#### Patient survey

Survey responses totalled 163, giving a response rate of 52% (*n* = 315). Of those, 155 (95%) had used the remote monitoring service. The majority were from the pilot hospital (119; 77%), over 50 years old (68%), female (77%) and white (82%). Almost half were in paid employment (47%) and education (2%).

**Figure 3.**
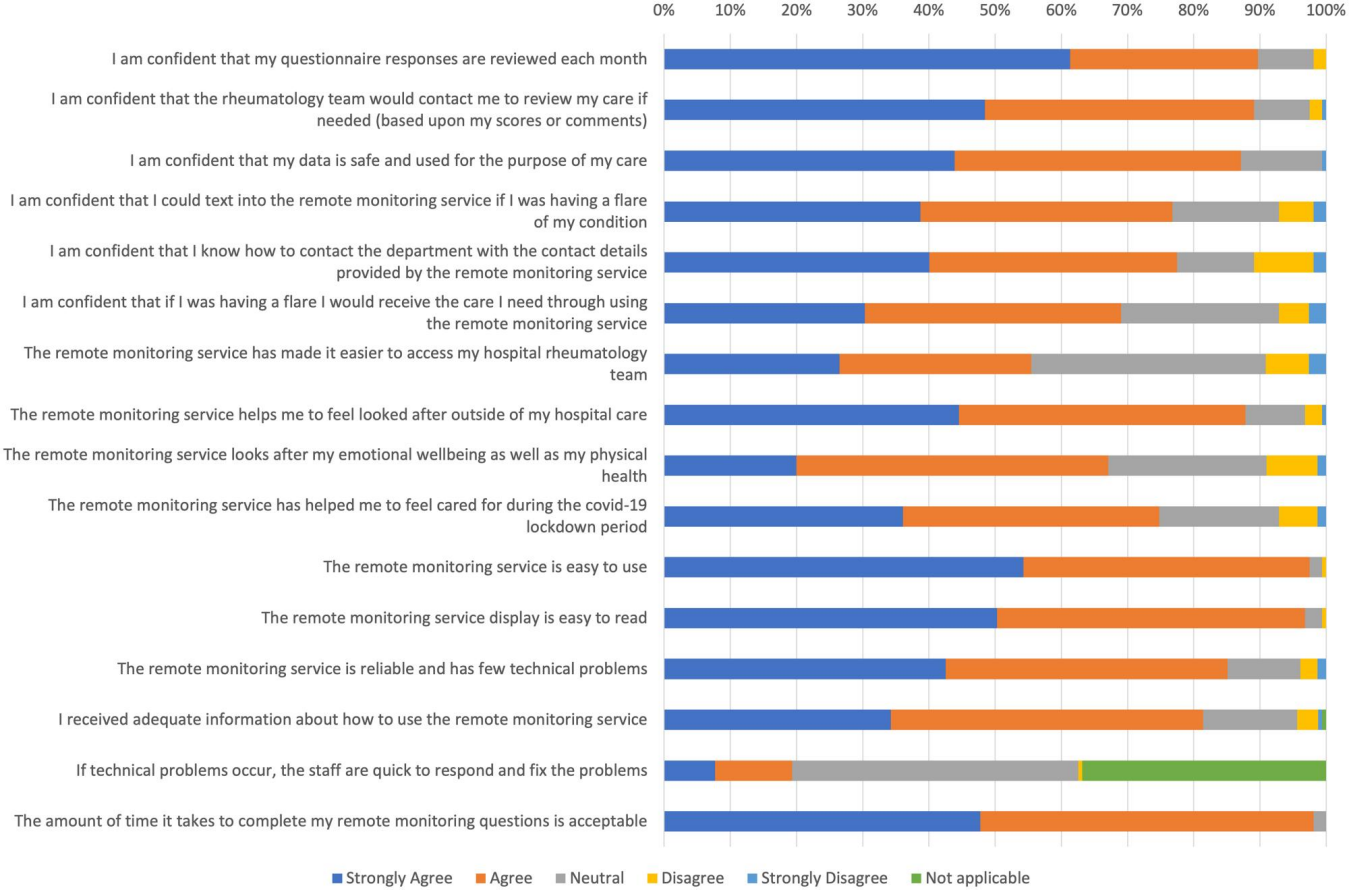
Patient survey results showing percentage of agreement for each statement.

Patients reported high levels of agreement (average 80%) across usability questions (Fig. 3). They had confidence in the service and felt it improved their access to rheumatology care. Many also felt the service helped them to feel looked after, emotionally as well as physically, outside of their hospital care—a feature that played an important role during COVID-19.

### Qualitative analysis

#### Patient interviews

Of the 22 patients interviewed, there were 19 females (86%) and 3 males (14%). Most were affiliated with the pilot hospital (18; 82%). Numbers from roll-out hospitals were low, but representative of those using the service. Six themes and 20 subthemes were developed for each domain of COM-B (Table 2).

**Table 2.** Patient interview results showing themes and subthemes, with example quotes, mapped across the Capability, Opportunity, Motivation, Behaviour (COM-B) framework

**Theme**	Subtheme	Quote
**Capability**		
Enhancing patient capability through the remote monitoring service opportunities	Technology skills: No special skills needed to use the service when interacting with technology using smart phones	‘I obviously use my smartphone every day. I'm quite happy to go on the internet, and to use it as a phone to use it, use all its facilities really … so it’s nothing new, it’s very straightforward’
	Psychological capability: Development of inner expertise on using the service metrics to correspond to the needs of their current health state	‘Once you get used to it coming in, you sort of get used to the scales and you get used to putting a number on the way you feel, yes piece of cake’
**Opportunity**		
Design of the remote monitoring service supports opportunities for engagement	Technical features: Patient interface with the service, e.g. text arrival timing supports completion	‘Very happy with that as well. I just think it makes life easier. I tend to have my phone with me most the time. When I wake up in the morning, it’s first thing I pick up to see what messages I’ve got or whatever. And if the text is there, I’ll do it very quickly and then get on with my day’
	Resource provision: The remote monitoring service provides opportunities for self-management, e.g. facilitates access to useful resources	‘14 years, it's quite hard to remember how you were. And so, it's quite nice to see that and you could see [in the graph] if there was a peak in pain, you know, like I say, I'm usually the same, I think that's helpful … it's good that they're colour coded. So you can see where you are. And you think, oh, am I more green, or am I a bit more red?’
	Healthcare access: The remote monitoring service provides opportunities for access to clinical care, e.g. to report a flare-up	‘Then there's a screen which asks you if you'd like to add anything about your experiences, or whether you're flaring, or what problems you've got, and then that presumably just get sent off and somebody reviews it and usually somebody comes back little comment, or sorry you were bad week, last week, hope you are resting better now, you know, those sorts of comments’
	Clinical interaction: Suggestions to increase opportunities for engagement in clinical practice, e.g. provide a downloadable graph of results	‘It would be quite nice to be able to get that little graph. Although, you know, I mean, if you can't, you can't, it doesn't matter. It's interesting for that moment … I think it might be useful for consultations … So you'd have that information, because it's quite hard when you've got a limited amount of time on the phone, and you want to get it all in. And you always forget things, always, even if you wrote it down and took it with you, you know, you always forget things. But if you were looking back at your own assessment of how things had been that might help that conversation’
Support to use the remote monitoring service	Ease of use: ‘Intuitive’ and ‘very self-explanatory’ remote monitoring service	‘It's very obvious what it is [the remote monitoring service]. So, I didn't feel like I necessarily needed it explained any further’.
	Patient space: No need to seek support from others in completing the remote monitoring questionnaire	‘I don't even think my family or friends know I get it [laughter] I've not mentioned it. I just do it’
	Patient autonomy: Promoting patients’ independence in having control of disease progress	‘For the most part ... I don’t talk about my condition that much, but my family is quite aware of it and will ask me quite regularly how are things going, and it’s actually really been quite nice to have the graph and data to show them to show how I’m doing really. So, every now and then I will discuss it with someone but for the most part I’m just sorting it out on my own’
Completing the questionnaire fits into patients’ routine	Ease of completion: Completing the questionnaire ‘straight away’	‘Within the hour usually, always within the day’
	Fit into routine: No barriers to completing the remote monitoring questionnaire	‘The only thing that would be doing it would be forgetting. Because obviously, there are other things going on in life and sometimes your rheumatoid arthritis isn't the highest thing on the list, it’s sort of bubbling along the bottom of your list of concerns because life goes on’
**Motivation**		
Automatic motivational processes	Resource provision: Existing relationship with RA services	‘You know, God, just people collecting numbers again, but I think, you know, if I'd stopped to think about it, then I would know that that's actually not my experience with Guy’s [hospital], because a few things have happened over the years that have kind of just let me know subconsciously that somebody is monitoring something somewhere’
	Physical convenience: Motivation to avoid hospital, e.g. previous medical trauma and Covid-19 anxiety; reducing unnecessary trips to the hospital	‘I quite like the remote access to GPs and doctors and hospitals and things. I think it's because I have a bit of a thing because I was in hospital for nearly two years when I was first diagnosed when I was little. And so, if I can avoid going to a hospital, I'm quite happy to do that really. That sounds a bit pathetic, but that's probably the reason’
	Re-assurance mechanism: The remote monitoring questionnaire acts as a way for patients to feel cared for	‘It was also like a bit of reassurance that I was doing this on a monthly basis and even if I didn’t have any appointment with the team or anything like that I felt that I was taking care of, for my condition and I think that was really positive, from a mental perspective’
Reflective motivational processes	Disease state view: The remote monitoring questionnaire acts as a way for patients to view their condition.	‘Because … the questions related to how did you feel last week, so I thought it would be better if it was, “How did you feel since the last Monitoring Service was done?”, so it’s more of a broader picture. During the time, in that week I’ll be fine but the previous weeks I might have experienced some pain, so I felt like it wasn’t capturing everything properly’
	Convenience: Motivation to avoid hospital, e.g. saving time and/or money for NHS and self	‘I suppose, apart from giving me support and information, it's also to release time for the rheumatology services, focus on people that really do need the help, and to prioritise who needs help and who is okay, for a non-face to face contact’
	Access to clinical team: Increased or easier contact with the RA team	‘Before the pandemic it was very, very difficult to get in touch with the rheumatology clinic. You could phone and nobody would answer the phone. And I think, probably, that was one of the reasons I thought it was quite a good idea’
	Patient autonomy: Improved self-monitoring and/or self-management	‘So, I think the way that I manage my condition is by understanding more about it as well. So … knowing when the flare up is severe enough to do something else about it and so having the ability to track it is involving me more in … care of the condition’
	Disease state comprehension: Data use and broader usefulness to RA team or NHS	‘It’s a personal monitoring service but at the same time you’re creating, adding to a database I assume where you can establish or see trends … I always try to respond to requests like that from the hospitals because I think it helps the doctors and everybody to create a database or whatever, which is always good’

NHS: National Health Service.

The remote monitoring service was regarded as easy to use and no special skills were needed. Patients’ psychological capability in using the service increased over time, as they became more familiar with the way it worked. Engagement with the RAID questionnaire was further supported by its ‘intuitive’ user-friendly design.

Patients reported that, by facilitating a greater understanding of their RA, the remote monitoring service provided opportunities to support self-management. Social opportunities were also created through prompting patients to book appointments in response to RAID scores or free text comments. This act gave patients ‘permission’ to make contact, and in doing so, alleviated fears of ‘wasting time’. Opportunities for access to clinical care were also provided. No major barriers to completing the RAID questionnaires were reported.

Major motivating factors were increased responsiveness and ease of contact with clinical teams. However, these were dependant on baseline levels of contact with clinical teams. The ability to submit free texts was a highlighted feature. Additional motivators included: increased self-awareness and/or self-management, feeling cared for, existing trusting relationships with clinical teams, and avoidance of hospital visits. COVID-19 was also cited by patients as a facilitator, as it normalized remote health monitoring, and intensified the desire to avoid hospital visits. Although use of remote monitoring service data in routine appointments was reported as desirable, it was not a significant motivating factor.

Conflicts in patients’ views were apparent, for example beliefs about usefulness of the service, which depended on using 4-weekly RAIDs to capture accurate information about patients’ disease activity. Some patients questioned this accuracy, for example when their disease fluctuated a lot. Conflicts also existed surrounding use of the remote monitoring service to cancel or postpone face-to-face appointments *vs* use of the service as supplementary to usual care. In addition, while most patients reported positive experiences, some felt the RAID prompts acted as an unwelcome reminder of their disease.

### Staff interviews

Sixteen staff were interviewed, which included individuals in leadership and clinical roles, as well as administrators. All hospitals were represented, with most participants recruited from the pilot hospital (11; 69%). Levels of staff engagement with the evaluation resembled their pattern of engagement with the service. Key findings were mapped across EPIS (Table 3 and Supplementary Table S3, available at *Rheumatology* online).

**Table 3.** Staff interview results showing categories, codes and key findings mapped across the Exploration, Preparation, Implementation, Sustainment (EPIS) framework

Phase	Construct	Category	Code	Key findings
Exploration	Inner context	Leadership	Involvement	Varying levels of involvement across sites from 2017 to 2020
			Needs assessment	Variable awareness of and involvement in needs assessment activities across sites, which were felt to focus on patient needs
Preparation	Innovation factors	Innovation characteristics	Development team	Digital product development team were supportive and engaged
			Digital platform	Many felt the platform was well-designed and user-friendly, but one individual felt it was not ready for clinician engagement
			Administrative support	The DPC has a central role in managing the remote monitoring service, which makes it easy to use. This ‘human factor’ also enables effective triaging of patient responses
			Service design	The remote monitoring service was well-designed for both staff and patients. Existence of a previous database laid important groundwork
		Fit to clinicians	Use alongside face-to-face appointments	Although the remote monitoring service should not replace face-to-face appointments, many felt it could be a useful complement and creates the potential to allocate and use face-to-face appointments more effectively. However, some questioned the necessity and value of the service
			Potential to support clinical decision making	Potential to support clinical decision making, as regular capture of PROM scores can provide a more objective representation of a patient's disease activity over time, but limitations exist
			Unmet expectations	Expectations of developing a ‘database’ for all patients not delivered
		Fit to patients	Patient characteristics	Suitable for most patients but not all, including those with more active disease, a secondary pain diagnosis, those with poor communication skills and who are unable or unwilling to use the internet. Some sociodemographic barriers may be more prevalent in certain contexts
			Fit into day-to-day routine	Remote monitoring service is quick, easy and non-intrusive for patients, *vs* burdensome, anxiety-inducing and an unwelcome reminder of disease
			Connection and safety	An alternative and potentially more effective line of communication for patients, which can offer a feeling of connection, safety and improved access to care. But worry that this could replace face-to-face care
			Promoting self-management or dependence	Offering patients more autonomy and empowerment, which can support self-management, *vs* encouraging overreliance on clinical services
		Fit to system		Blending face-to-face and remote working is important for future service provision
		Adaptations		Considerations made to adapt service to suit local needs
	Inner context	Leadership	Leadership characteristics	Passionate and engaged multidisciplinary leadership team
			Teamwork and collaboration	Desire for shared understanding and collaboration, but concerns and feedback not always taken on board
			Communication	Communications were varied, focused on the patient perspective, and served as reminders, to report progress and elicit feedback
			Advocates	The varied success of appointed champions was bolstered by the emergence of unexpected advocates
		Clinician factors	Clinician characteristics	Facilitators include clinical and digital acumen, familiarity with remote monitoring technology, but clinical experience may dictate levels of engagement
			Teamwork and collaboration	Local teamworking efforts seen as a facilitator
			Attitude towards innovation	Fear of increased workload, loss of ‘control’ over patients, and lack of ‘confidence’ in the remote monitoring service
			Readiness for change	Potential lack of readiness for change
	Outer context	Leadership ties		Clinical Lead liaised with commissioners and senior boards
		Inter-organizational environment		Similar platforms may compete for clinicians’ attention
		Climate		The coronavirus pandemic created favourable implementation conditions, as the remote monitoring service helped meet evolving needs. However, these advantages were offset by barriers introduced by the pandemic
	Bridging factors			NHS Innovation Team provided support and funding
Implementation	Engagement			Staff engagement was variable over time and greatest at the pilot site. Recent increases attributed to innovation adaptations
	Innovation factors	Innovation characteristics	Digital platform	Several limitations mentioned, including lack of automatic start-up and visual prompts, log-in difficulties and lack of interoperability with existing programmes
			Administrative support	Good admin support, for example with recruitment, monitoring and clinical escalation, makes the service straightforward for clinicians to use
			Training	Staff spoke positively about training, with some informally adopting a ‘train-the-trainer’ approach. However, the requirement for training to permit log-in access also acted as a ‘limiting factor’ and discouraged engagement of the innovation
		Fit to clinicians		Not being able to identify which patients are onboard the service acted as a barrier
		Adaptations		Adaptations to address staff needs received mixed reviews. These included increasing the availability of training, making the platform more user-friendly, relaxing patient eligibility criteria and refining triaging processes. Shifting the responsibility of patient management towards clinicians was particularly controversial
	Inner context	Leadership	Leadership characteristics	Clinician involvement brought new insights, but lack of clear leadership seen as a barrier
			Communication	Communications around service promotion and training facilitated clinician engagement, but others commented that the strategy was at times unrealistic and misguided
			Evaluation	Lack of evaluation activities
		Organizational support		Lack of tangible organizational support
		Clinician factors	Attitude towards innovation	Remote monitoring service not seen as a priority in busy, time-pressured clinics
		Barriers at roll-out sites	Leadership—communication and champions	Poor leadership engagement, for example lack of a regular ‘in-person’ presence and effective champions
			Organizational characteristics	A central approach to patient management at roll-out sites was challenging. Plus, potential lack of appreciation of contextual differences
			Clinician factors—attitude towards innovation	Distrust and feeling like unequal partners
	Outer context	Inter-organizational environment		Difficulties surrounding patient management arising from a centralized approach based at the pilot site
		Climate		The coronavirus pandemic reduced clinicians’ capacity to implement the remote monitoring service, through reduced manpower, disruption to clinical services and changing patient needs
Sustainment	Feasibility			Wider scale up could be feasible, especially in smaller trusts
	Innovation factors	Innovation characteristics	Digital platform	Ensuring the system has inbuilt flexibility to respond to local and evolving needs, and addressing current unmet needs, in particular the lack of integration with other key platforms
			Administrative support	Administrative support should be increased in line with service expansion, feature at all relevant clinical sites, and include properly trained staff, information technology support and improved infrastructure
			Service design	Improving patient guidance to promote self-management, to ensure patient safety and help manage workloads
		Fit to clinicians		Important to prioritise clinician needs alongside patient needs
	Inner context	Leadership	Leadership characteristics	Strong leadership with good listening skills needed
			Communication	Effective communications strategy that includes regular stakeholder engagement and face-to-face time
			Evaluation	Requirement for comprehensive and transparent evaluation activities
		Organizational support		Need for robust organizational support
		Clinician factors	Readiness for change	Need for culture change and consideration of readiness for change

DPC: Digital Pathway Coordinator; PROM: Patient Reported Outcome Measure.

Exploration was the least prominent phase. Significant overlap between ‘preparation’ and ‘implementation’ was evident, and examination of facilitators and barriers to implementation across these phases revealed a range of views. Many felt the service was easy to use and provided a good complement to face-to-face appointments. This blended approach was seen as important for future service provision. Most staff felt the service represented a good fit for clinicians. However, this was tempered by issues related to functional limitations, including lack of interoperability with existing platforms, negative attitudes, for example fear of increased workload, loss of ‘control’ over patients and lack of ‘confidence’ in the service, and resistance to change. Adaptations to optimize the service received mixed reviews.

Most staff felt the service was good for patients, promoting autonomy, empowerment, and facilitating patient-initiated follow-up. However, it was agreed that the service would not be suitable for some, for example those with more active disease, a secondary pain diagnosis, poor communication skills and those unable or unwilling to use the internet.

Administrative support and leadership, particularly engagement strategy, were elements that featured throughout staff interviews. Views related to these were mixed, with tangible organizational support felt to be lacking. COVID-19 had dual effects, initially creating favourable conditions, but later preventing in-person support and disrupting services. Some barriers were specific to implementation at roll-out hospitals. These included poor leadership engagement with teams at roll-out sites, varying practices at different sites and distrust towards other organizations. Patient populations with different characteristics and needs may also play a role in varying engagement between hospitals.

Factors important for sustainability included addressing current unmet needs, especially integration with the patient electronic health record, and responsiveness to evolving needs. Although putting patient needs at the centre of the service was seen as valuable, equally prioritizing clinician needs in relation to implementation within services was also highlighted. Staff stressed the importance of strong leadership with a nuanced approach to understanding local challenges, and patient safety. Wider scale-up of the service was thought feasible by some. However, caution was expressed around implementation in larger organizations which may experience more logistical challenges, such as communication with clinical teams. Clear and transparent communication, good stakeholder engagement, a strong evidence base, and presence on governance agendas would be instrumental.

## Discussion

This study evaluated patient and staff experiences and implementation outcomes in the context of the scale up of a remote monitoring service for patients with RA (in remission or with low disease activity) attending outpatient clinics across six NHS hospitals in London, UK. The service was built with user-centred design methodology and extensive stakeholder involvement, with a view to optimizing engagement [17]. As far as we are aware, this is the first description, and subsequent evaluation, of such a service across multiple organizations.

High levels of patient engagement with the service were evident and this was sustained over a significant 34-month period. This demonstrates the success of the patient-centred platform design approach. COM-B was useful in providing a deeper understanding of the factors that drove this. Ease of use and improved access to care were features that stood out. Importantly, feelings surrounding access to care were not only dependant on patients’ baseline level of contact with their service but were also likely to be influenced by COVID-19. Patient engagement was also driven by opportunities that supported increased self-management and, ultimately, taking more control over their condition. These findings are in line with recent evidence that suggests that patients with RA may be amenable to remote monitoring of PROMs if it facilitates useful communication with healthcare providers, as well as providing access to reliable information about RA [37].

EPIS provided a useful temporal lens to understand the implementation process and therefore to identify barriers and facilitators at different phases. In contrast to patients, staff views were more mixed, with some coherence and contradiction. Factors related to the service itself, leadership, stakeholder engagement and organizational support were recurrent themes influencing facilitation.

Consistent with the evidence review, clinicians agreed that the remote monitoring service offers the potential to bring valuable benefits both to clinical management, for example by facilitating improved allocation of appointments, and to patients, for example through empowerment. Caution around potential negative impacts to patients was expressed, for example being burdensome and anxiety-inducing, and warrants further study. Similar concerns have been raised elsewhere [29]. Further work is required to ensure that the service does not exacerbate existing health inequalities or digital exclusion.

Clinicians found the service easy to use but limitations, particularly around functionality, need to be addressed to improve sustainability and potential gains. For example, the potential value of integration with existing platforms, in particular the patient electronic health record, was emphasized—this has been highlighted elsewhere [9] and points to some of the challenges of developing innovations within resource restricted NHS settings, and of piloting minimally viable products as a proof of concept in routine practice settings [17]. Negative attitudes, for example fear of increased workload, were also prominent. This contrasts with patient data, which could suggest that clinicians’ needs were not taken into account to the same level as those of patients’ [17]. Indeed, exploration was the least prominent phase among staff and appeared to prioritize the patient perspective. This will be important to address in future, as clinicians are also key users of the service. Additional barriers specific to implementation at roll-out sites were evident, which may explain why clinician engagement was poorest here. Attempting ‘too much, too soon’ (Clinical Lead, evaluation workshop) was likely to be a contributing factor, but accelerated roll-out to meet the challenges of COVID-19 was a strong motivator.

### Strengths and limitations

The evaluation has limitations. A pragmatic approach was used to ensure the study could rapidly identify and incorporate lessons learned and maximize benefits to patients and other stakeholders. As such, there were deviations from the early protocol—namely, the scope of the subsequent evaluation was more restricted. For example, we did not conduct an exploration of service outcomes or an in-depth feasibility analysis. This was not a controlled evaluation, and therefore definitive conclusions about the relationship between observed or self-reported outcomes and the remote monitoring service cannot be made. Data were provided by staff and patients predominantly from the pilot hospital, and engaging with staff at roll-out hospitals proved challenging. This represents a limitation and detailed understanding warrants further study. However, it suggests that implementation beyond the pilot hospital was poor. Conducting the study at a time still very much affected by COVID-19 also had implications, for example, emphasis was placed on electronic rather than face-to-face interviewing approaches, which are preferable for establishing rapport [38]. The unique set of circumstances introduced by COVID-19 also limits the transferability of the findings to a COVID-19-free context. The evaluation also has several strengths—including the use of well-established implementation theory, multiple sources of data to allow triangulation, and the multi-stakeholder evaluation team.

## Conclusions

Patients were overwhelmingly positive about the remote monitoring service, and engagement levels remained high. Staff views were more divergent. Barriers specifically active at roll-out sites may explain low levels of clinician engagement beyond the pilot hospital. Importantly, results suggest that equal levels of patient and staff engagement are required for sustainability of the service. Patients and staff generally felt that the service was acceptable and could potentially lead to improved patient-centred care, clinical management and use of clinician capacity. Together, these findings further our understanding of the implementation challenges to scaling remote monitoring interventions for patients with RA (in remission or with low disease activity) in routine care settings, provide valuable insight into clinician factors influencing implementation—and are potentially applicable to similar remote monitoring interventions in the context of long-term care.

## Supplementary Material

keae112_Supplementary_Data

## Data Availability

The data underlying this article will be shared on reasonable request to the corresponding author.
