# Supplementary material for: Remote monitoring of patients with rheumatoid arthritis in a low disease activity state: a mixed methods evaluation across six hospitals in London, UK
Source: Rheumatology (Oxford). 2024 Feb 24;63(10):2721–33. doi: 10.1093/rheumatology/keae112 (PMC11443016; doi:10.1093/rheumatology/keae112)
Supplement: keae112_Supplementary_Data [file keae112_supplementary_data.docx]

**Supplementary material**

1. Supplementary Data S1. Eligibility criteria for onboarding patients onto the remote monitoring (RM) service.
2. Supplementary Figure S1. Overview of the remote monitoring (RM) service pathway.
3. Supplementary Figure S2. Flowchart illustrating the process of sending Rheumatoid Arthritis Impact of Disease (RAID) score requests to patients via the remote monitoring (RM) service.
4. Supplementary Data S2. Survey of patient views.
5. Supplementary Data S3. Semi-structured interview guides.
6. Supplementary Table S1. Extracted data from the rapid evidence synthesis review.
7. Supplementary Figure S3. Rheumatoid Arthritis Impact of Disease (RAID) score completion rates in relation to the number of requests sent.
8. Supplementary Table S2. Average Rheumatoid Arthritis Impact of Disease (RAID) score completion rates according to cumulative time onboard the remote monitoring (RM) service.
9. Supplementary Table S3. Staff interview results showing categories, codes, key findings and example quotes mapped across the Exploration, Preparation, Implementation, Sustainment (EPIS) framework.
10. Supplementary Data S4. Lay summary.

**Supplementary Data S1. Eligibility criteria for onboarding patients onto the remote monitoring (RM) service.** DAS28-CRP (28-joint Disease Activity Score with C reactive protein); RAID (Rheumatoid Arthritis Impact of Disease)

Patient were deemed eligible if they met the following criteria:

- Patient has a smart phone
- Patient and/or carer has sufficient written English language skills

Patient meets either criteria 1 or 2:

Criteria 1: DAS28-CRP ≤3.2 AND at least one of first 3 RAID scores ≤ 4

Criteria 2: DAS28-CRP unknown OR clinician agreed disease remission OR clinician agreed low disease activity AND at least one of first 3 RAID scores ≤ 2

**Supplementary Figure S1. Overview of the remote monitoring (RM) service pathway.** Eligible patients are onboarded onto the remote monitoring (RM) service after discussion and consent at their routine outpatient appointment. Monthly patient reported outcome measures (PROMs) and text messages are monitored by a Digital Pathway Coordinator (DPC), working closely with clinicians. Patients are triaged into one of three pathways. Ineligibility or ‘opting out’ triggers a return to standard care.


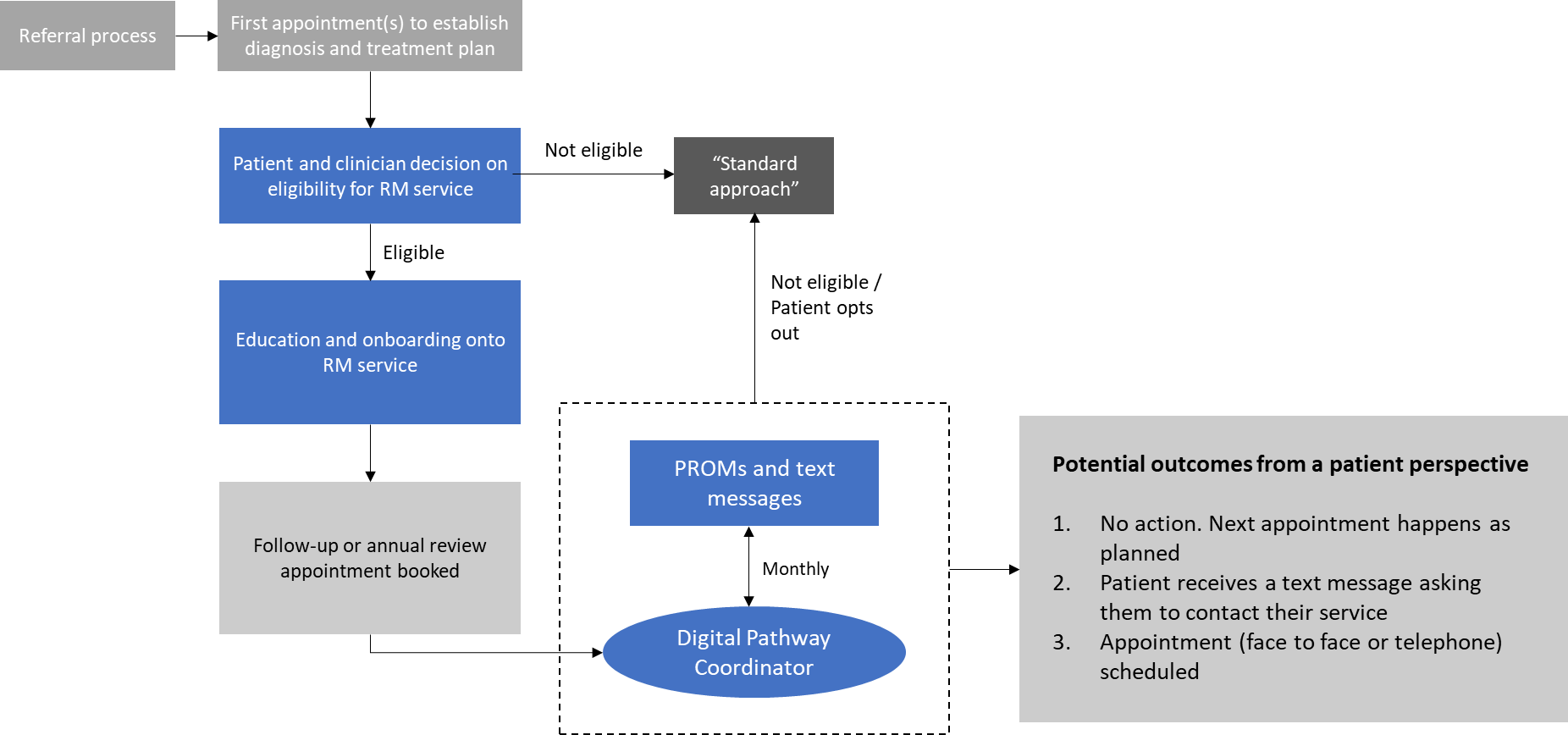


**Supplementary Figure S2. Flowchart illustrating the process of sending Rheumatoid Arthritis Impact of Disease (RAID) score requests to patients via the remote monitoring (RM) service.**

**
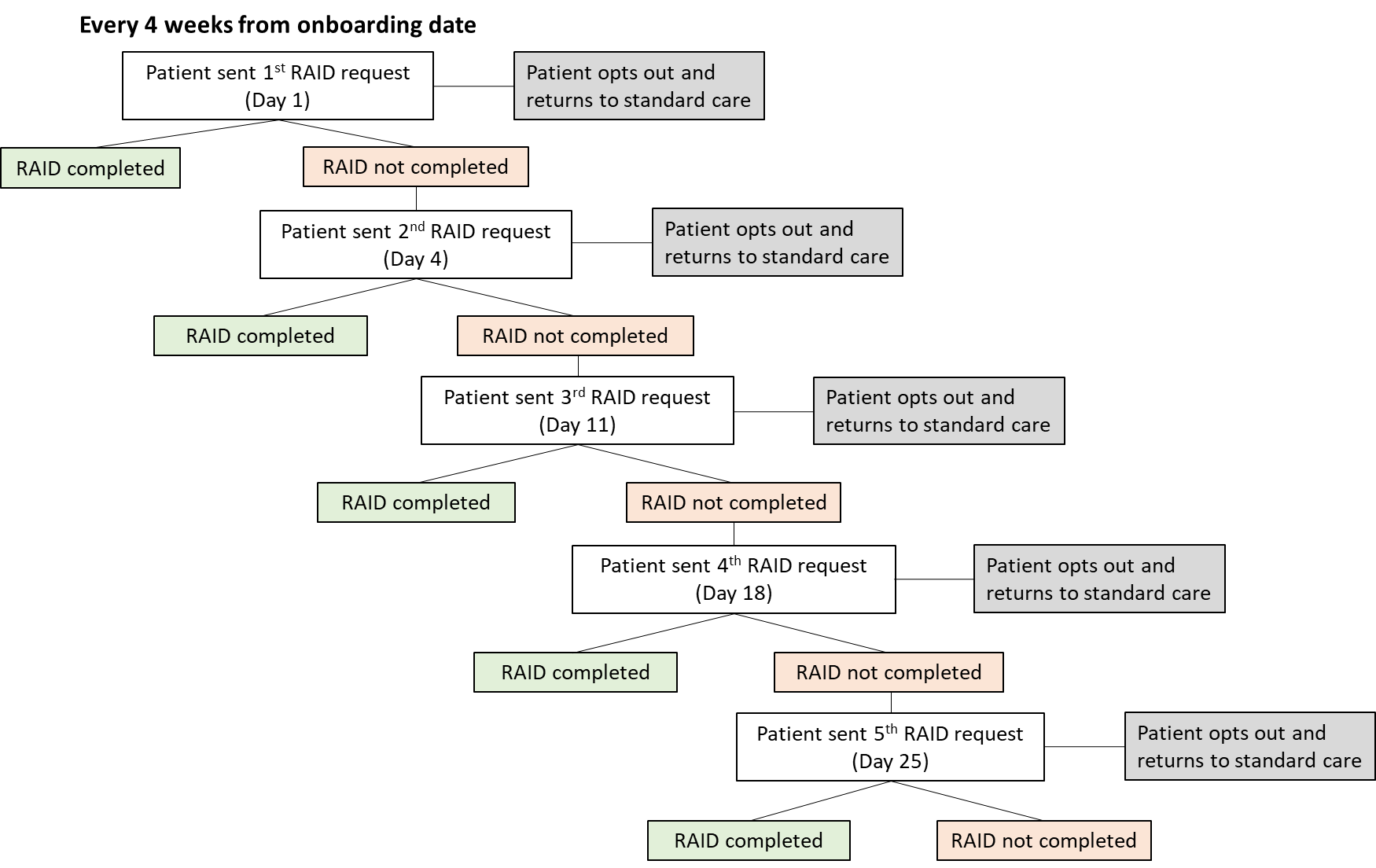
**

**Supplementary Data S2. Survey of patient views**

* Required

1. Please tell us which hospital provides your rheumatology care: *

- GSTT - Guy's and St Thomas' Hopsital
- KCH - King's College Hospital
- PRUH - Princess Royal University Hospital (Orpington)
- QEW - Queen Elizabeth Hospital
- QMS - Queen Mary's Hospital
- UHL - University Hospital Lewisham
- Another hospital

1. Please tell us the name of the hospital that provides your rheumatology care: *
2. This survey is about a service that hospitals in south east London offer their patients who receive care from a rheumatology outpatient clinic. Patients who use this remote monitoring service receive a text message every four weeks asking them to complete a series of questions on their phone about their symptoms and how they are coping. Have you used this service? *

- Yes
- No
- Not sure

**Your experience of the remote monitoring service**

1. Please tick the box that most closely represents your view *

|  | Strongly agree | Agree | Neutral | Disagree | Strongly disagree |
| --- | --- | --- | --- | --- | --- |
| I am confident that my questionnaire responses are reviewed each month | 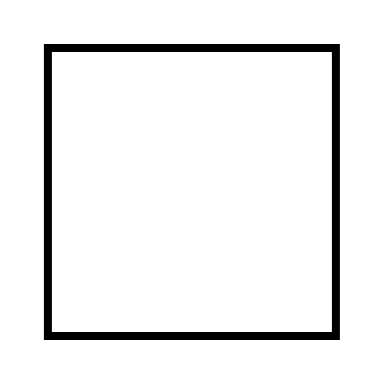 | 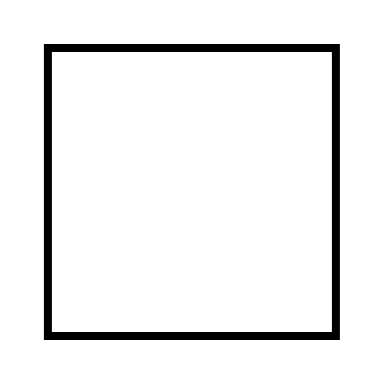 | 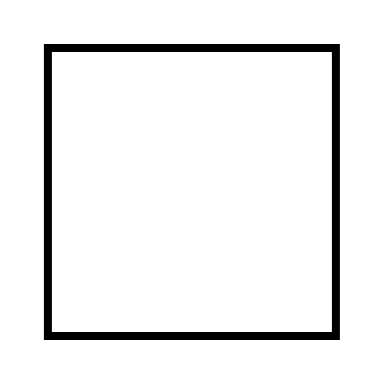 | 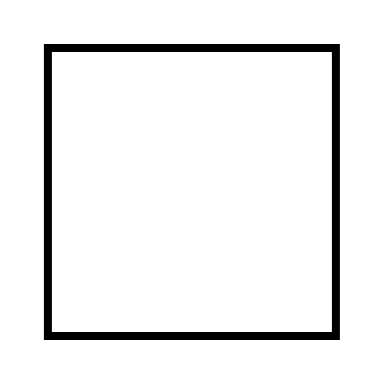 | 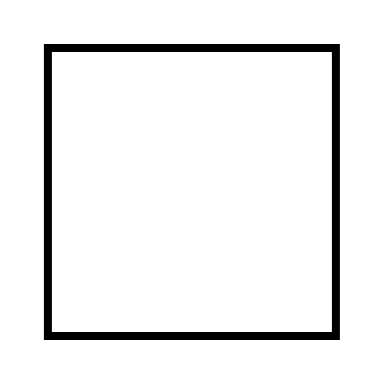 |
| I am confident that the rheumatology team would contact me to review my care if needed (based upon my scores or comments) | 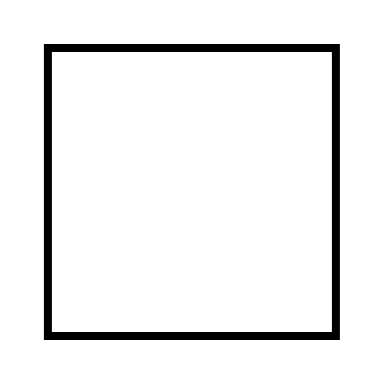 | 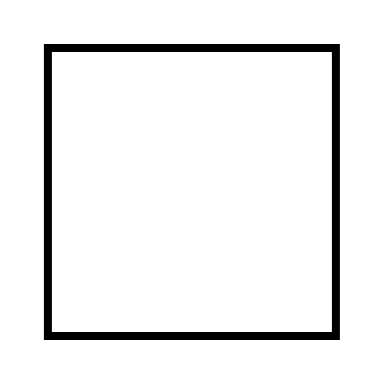 | 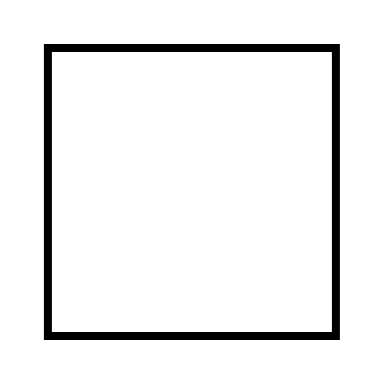 | 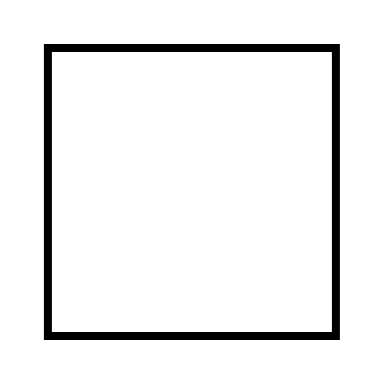 | 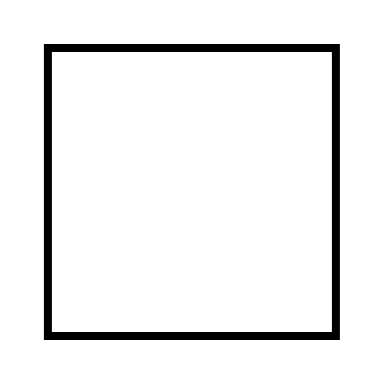 |
| I am confident that my data is safe and used for the purpose of my care | 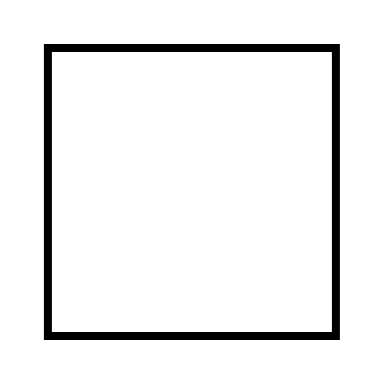 | 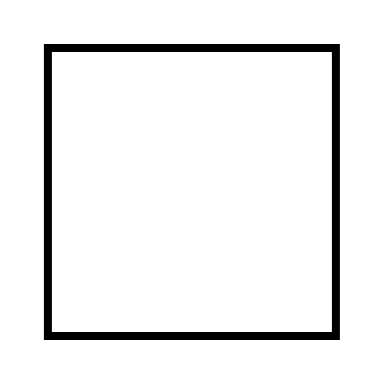 | 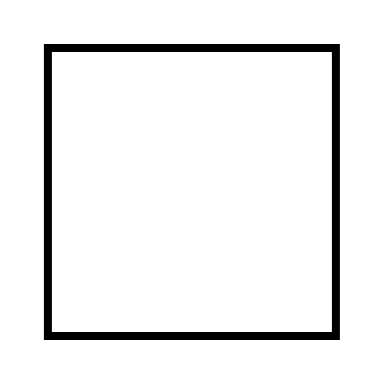 | 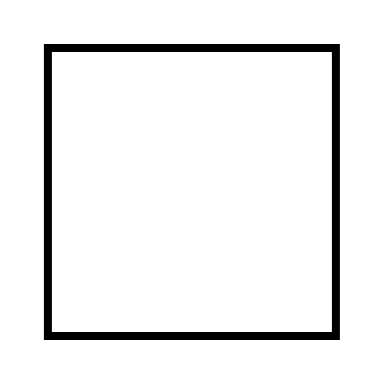 | 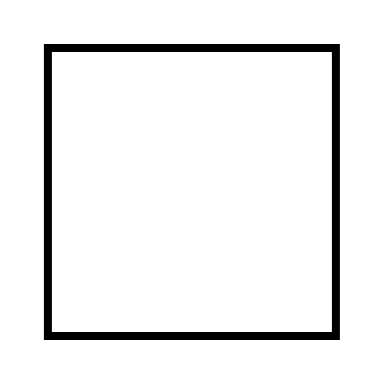 |
| I am confident that I could text into the remote monitoring service if I was having a flare of my condition | 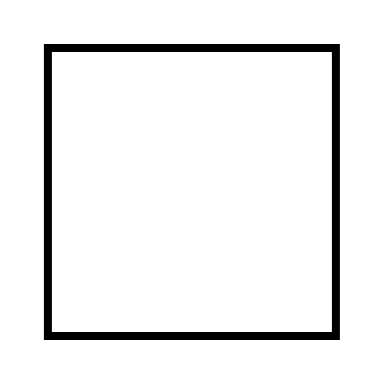 | 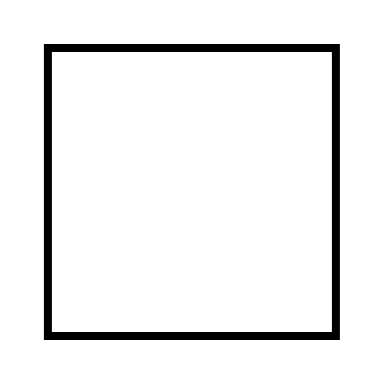 | 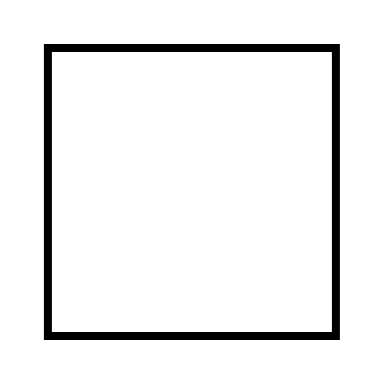 | 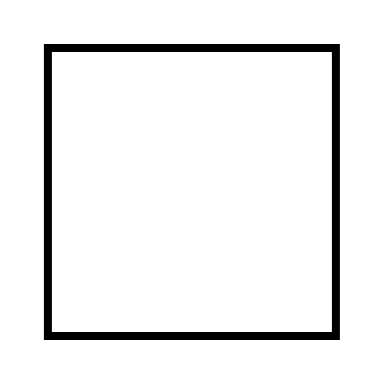 | 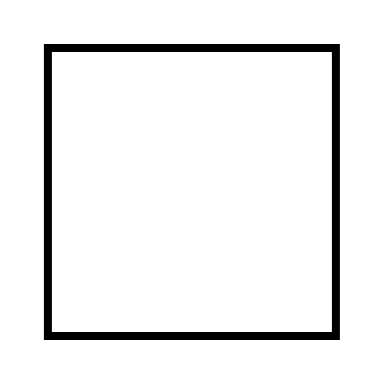 |
| I am confident that I know how to contact the department with the contact details provided by the remote monitoring service | 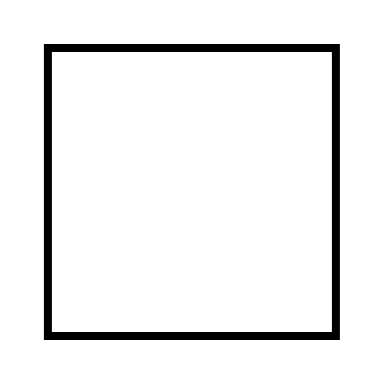 | 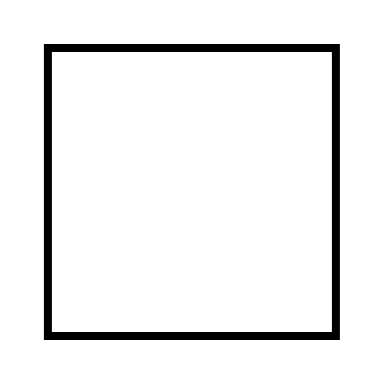 | 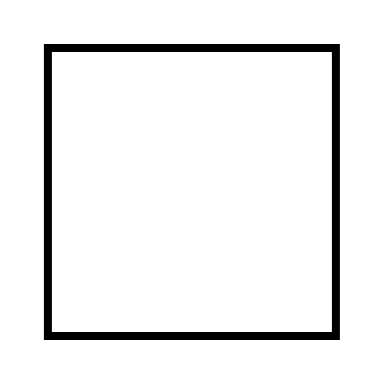 | 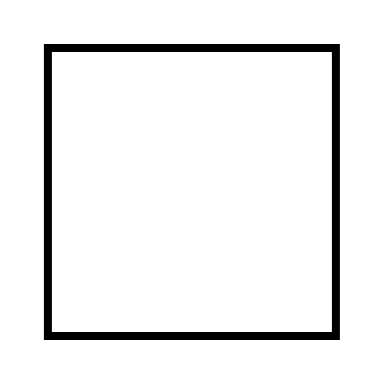 | 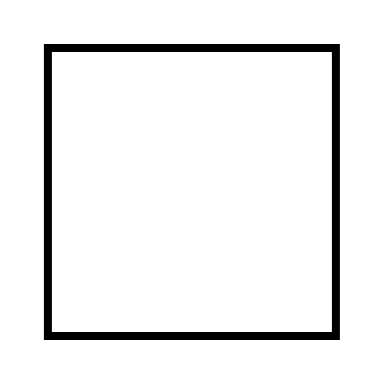 |
| I am confident that if I was having a flare I would receive the care I need through using the remote monitoring service | 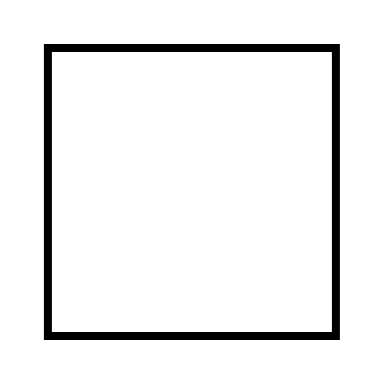 | 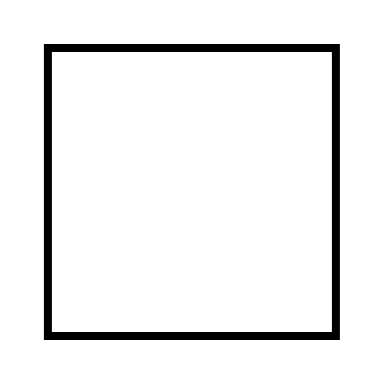 | 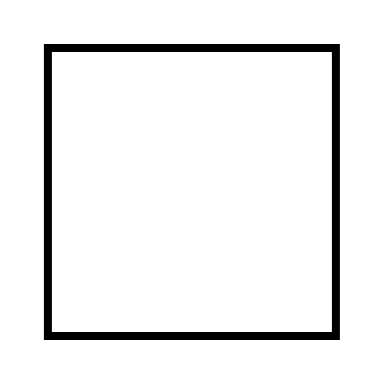 | 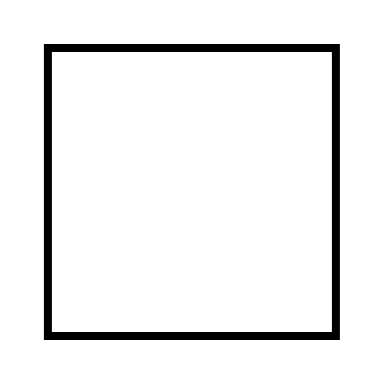 | 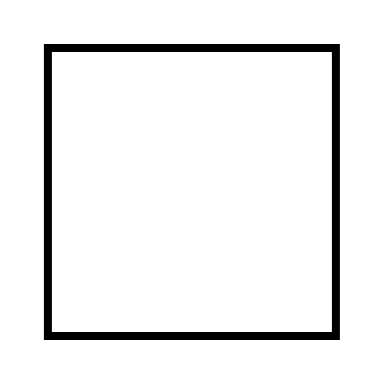 |
| Using the remote monitoring service has helped me to manage my flares better than before I started using this service | 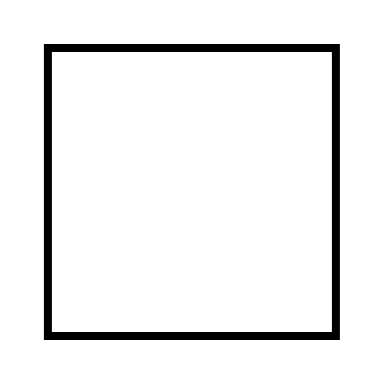 | 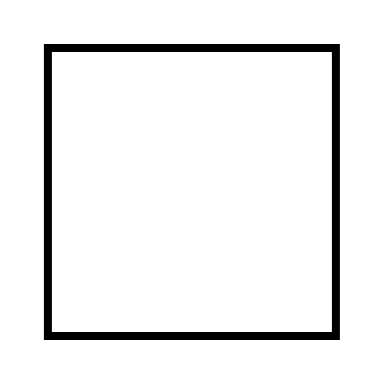 | 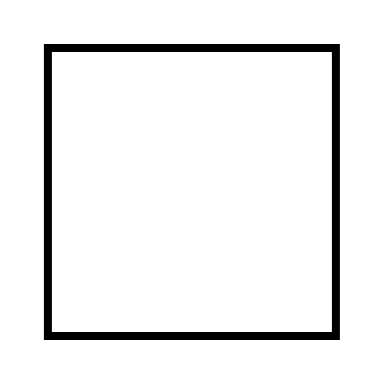 | 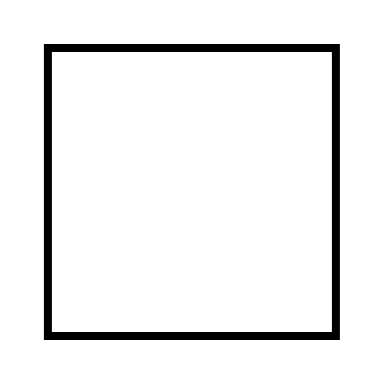 | 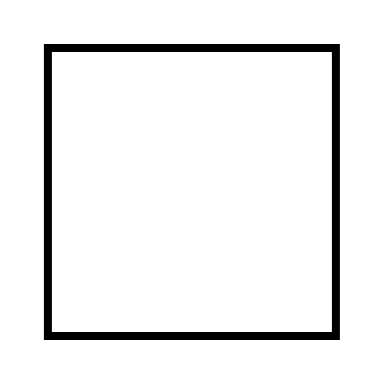 |
| The remote monitoring service has made it easier to access my hospital rheumatology  team | 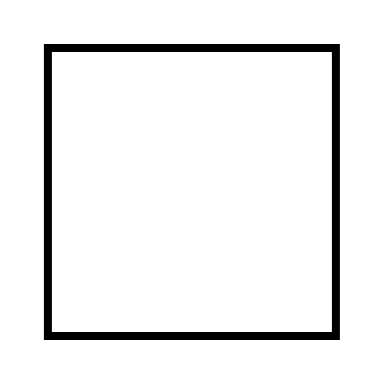 | 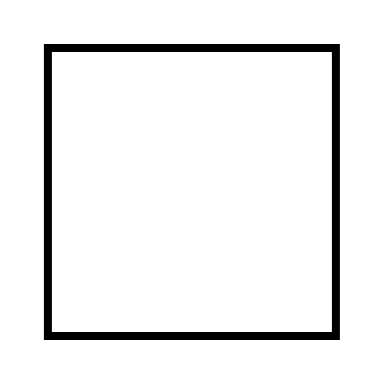 | 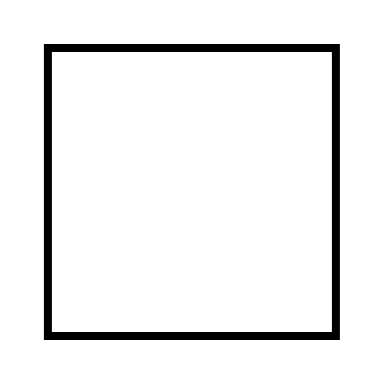 | 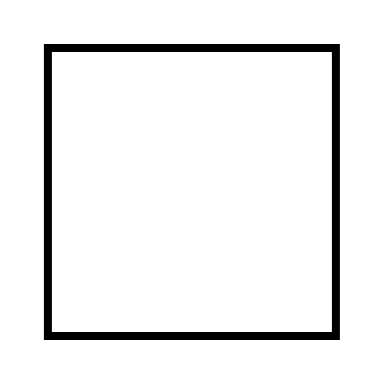 | 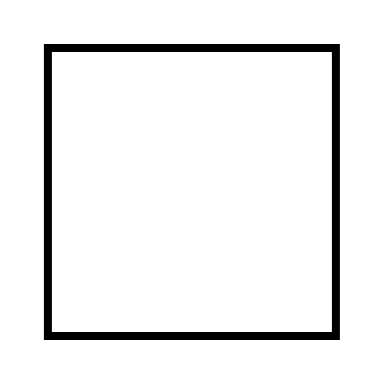 |

1. Please tick the box that most closely represents your view *

|  | Strongly agree | Agree | Neutral | Disagree | Strongly disagree |
| --- | --- | --- | --- | --- | --- |
| The remote monitoring service helps me to feel looked after outside of my hospital care | 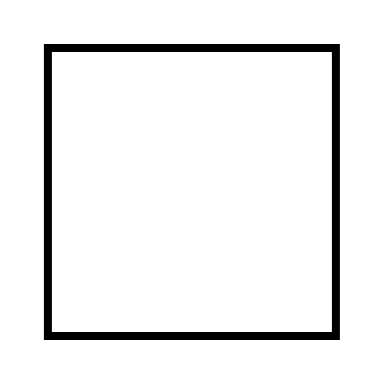 | 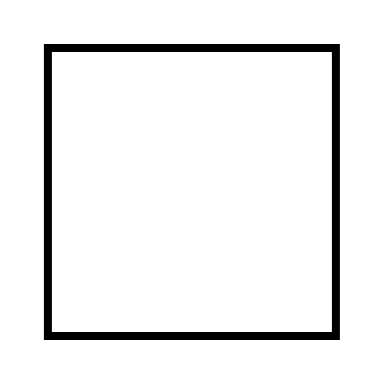 | 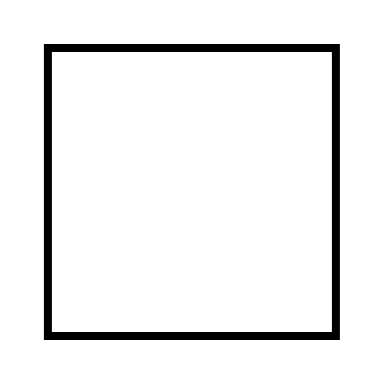 | 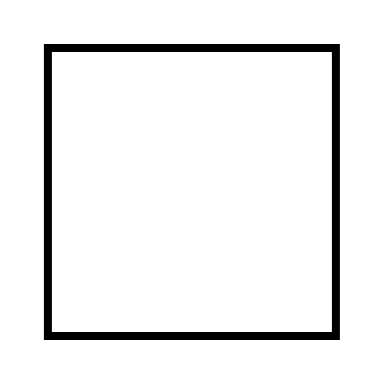 | 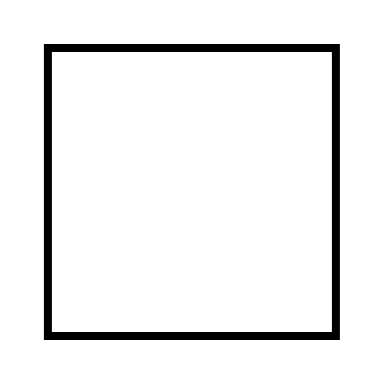 |
| The remote monitoring service looks after my emotional wellbeing as well as my physical health | 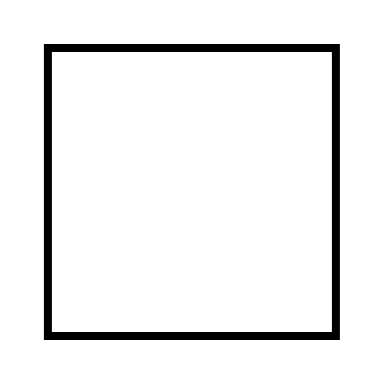 | 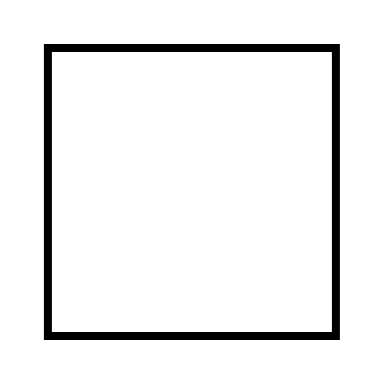 | 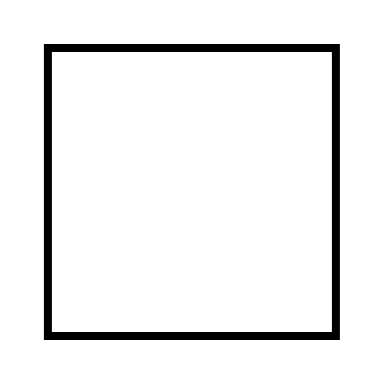 | 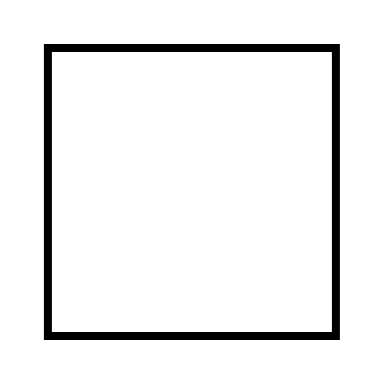 | 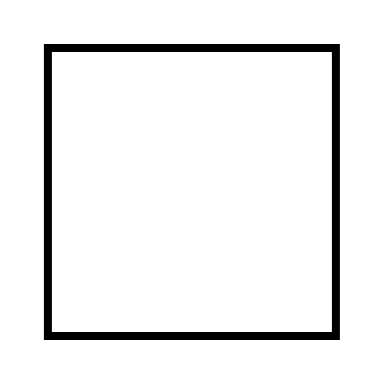 |
| The remote monitoring service has helped me to feel cared for during the covid-19 lockdown period | 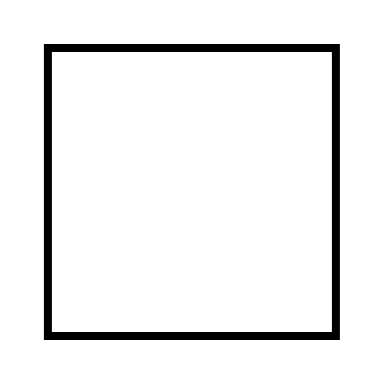 | 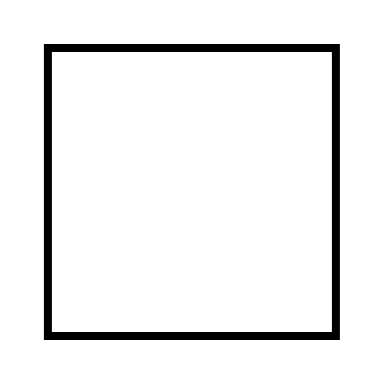 | 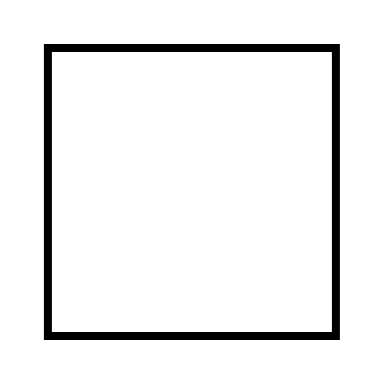 | 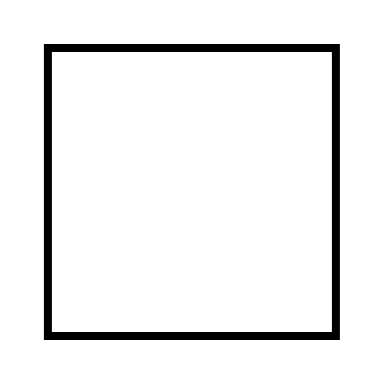 | 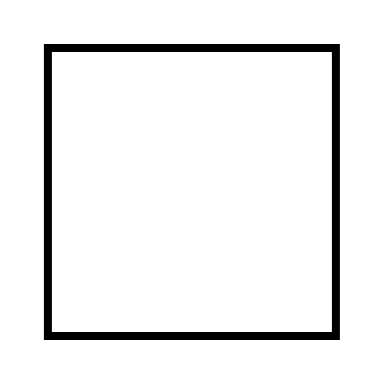 |
| My rheumatology appointments are not always when I need them | 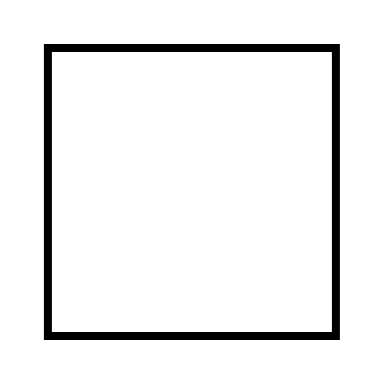 | 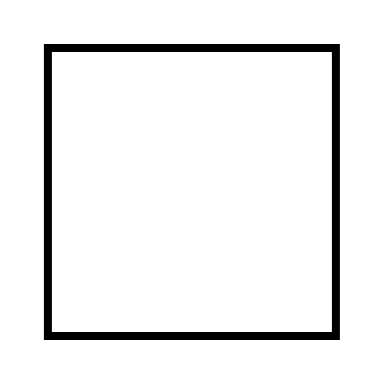 | 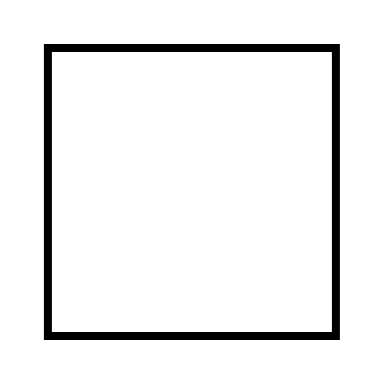 | 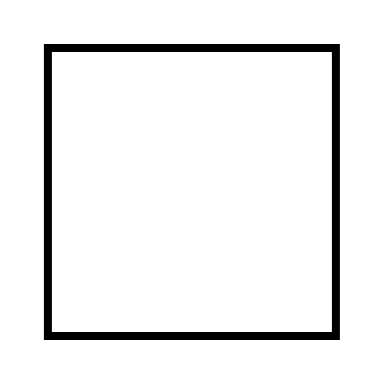 | 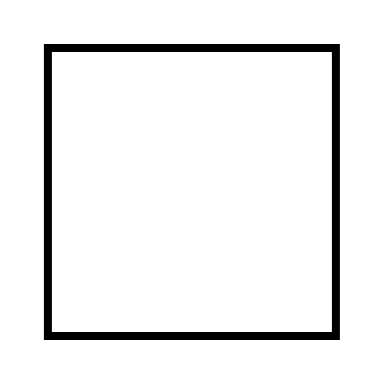 |
| I would consider deferring an  appointment if I was well as my appointment approached | 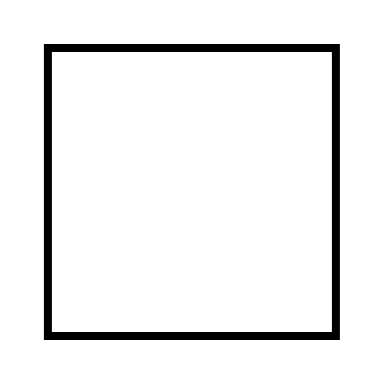 | 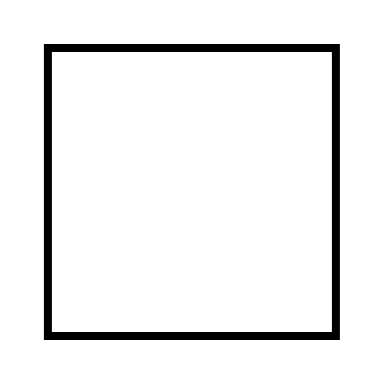 | 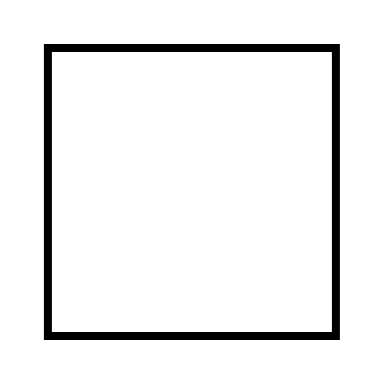 | 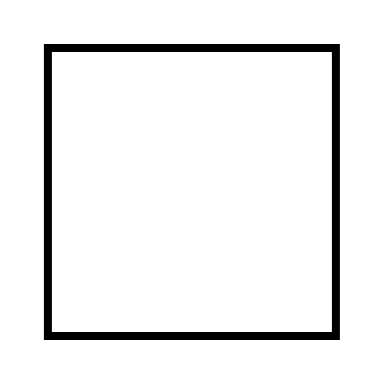 | 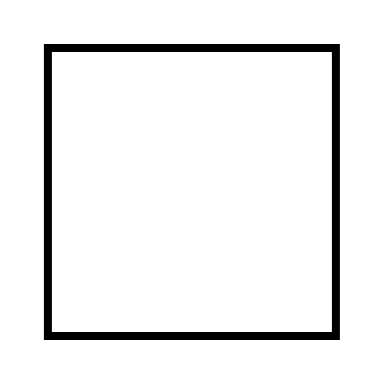 |
| I feel confident I would be offered an  appointment when I needed it e.g if I was flaring | 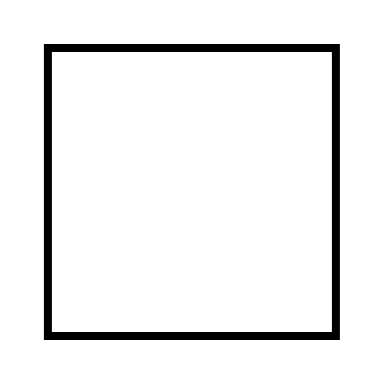 | 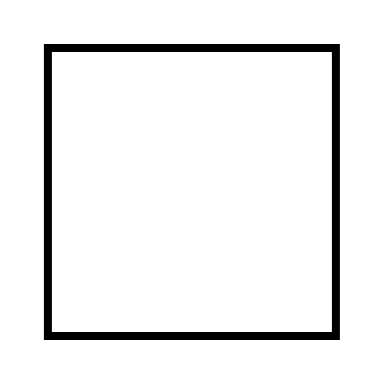 | 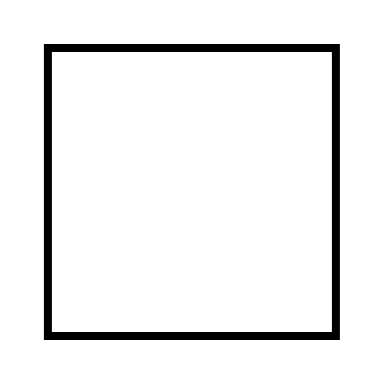 | 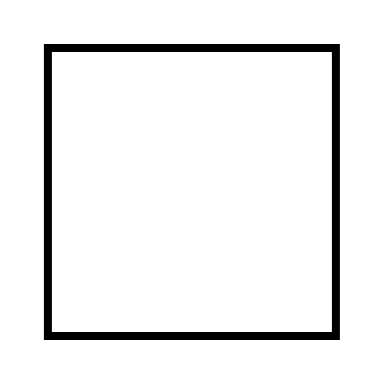 | 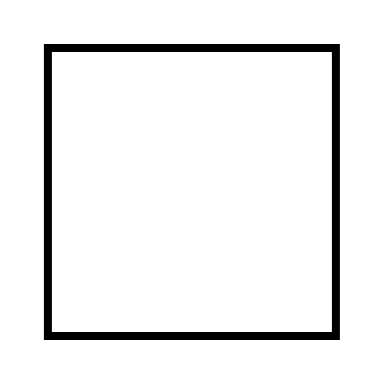 |
| A telephone consultation is an  acceptable alternative to attending a hospital appointment | 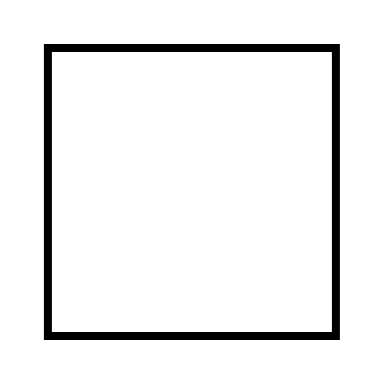 | 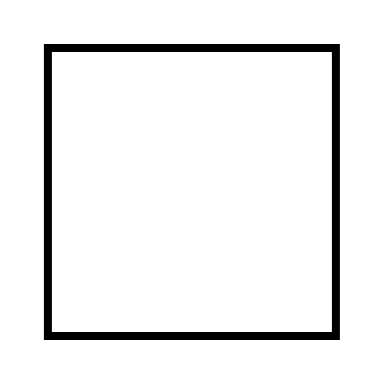 | 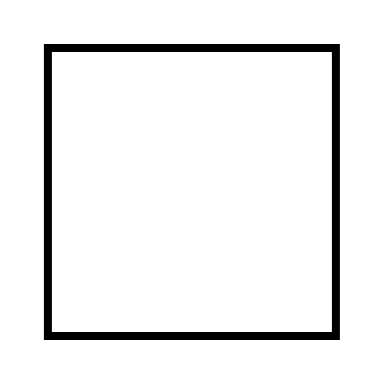 | 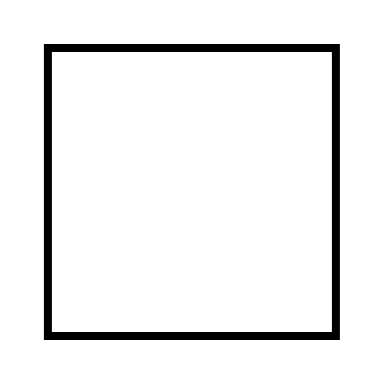 | 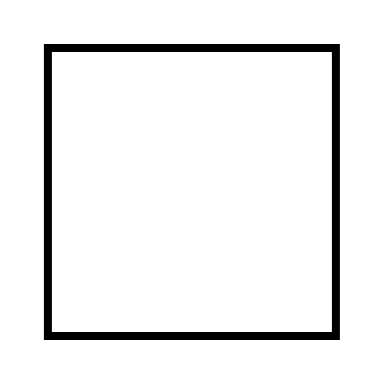 |
| A video consultation is an acceptable alternative to attending a hospital appointment | 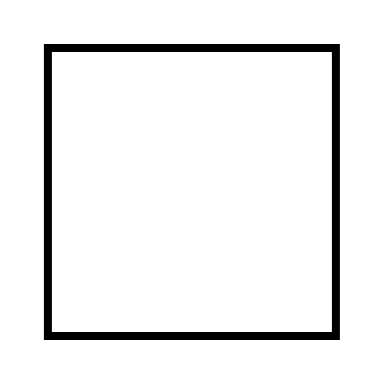 | 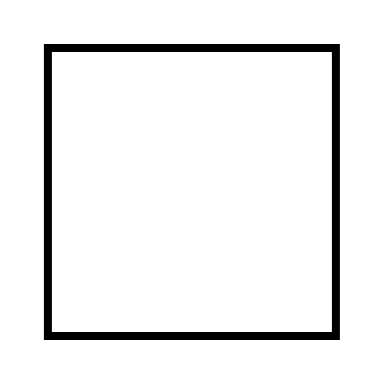 | 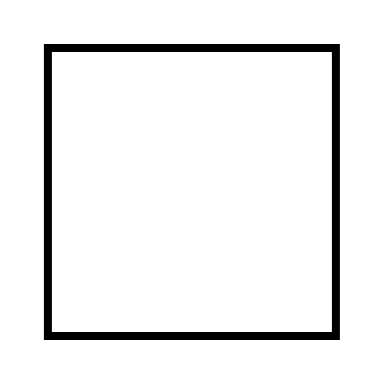 | 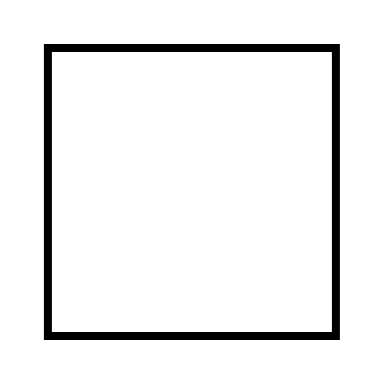 | 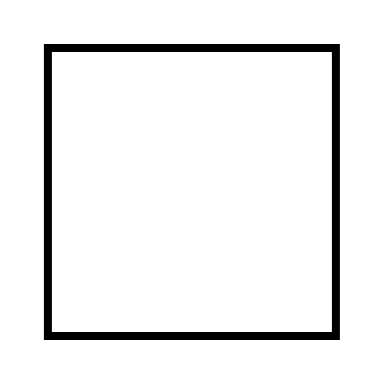 |
| Since I have been using the remote monitoring service, I have had fewer unnecessary appointments at the outpatient clinic | 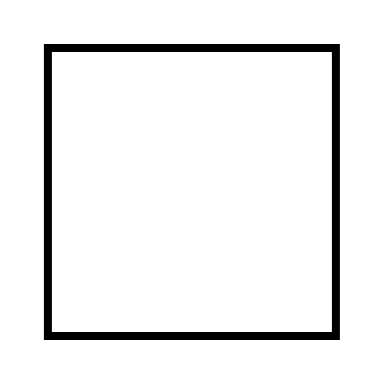 | 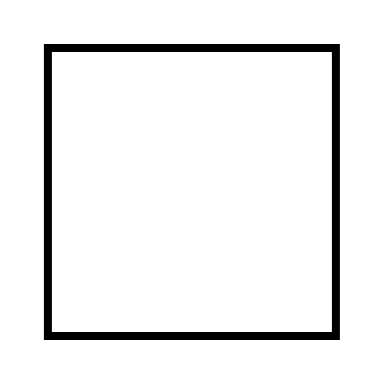 | 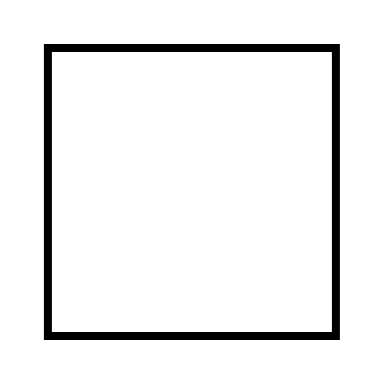 | 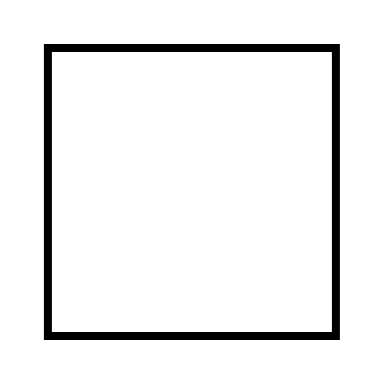 | 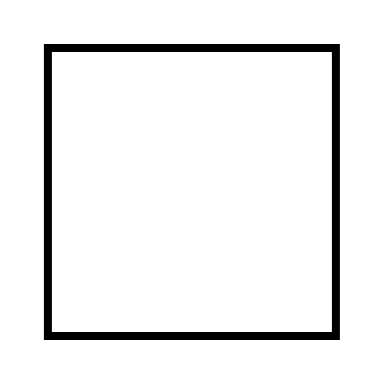 |

1. The next few statements are about ease of use, please tick the box that most closely represents your view *

|  | Strongly agree | Agree | Neutral | Disagree | Strongly disagree |
| --- | --- | --- | --- | --- | --- |
| The remote monitoring service is easy to use | 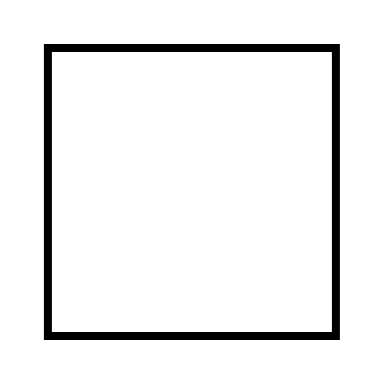 | 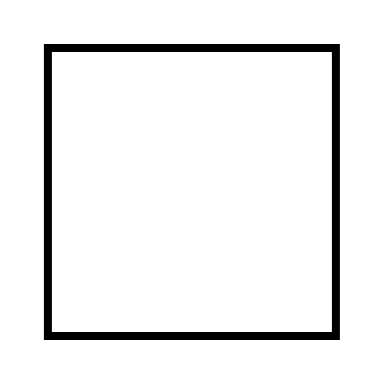 | 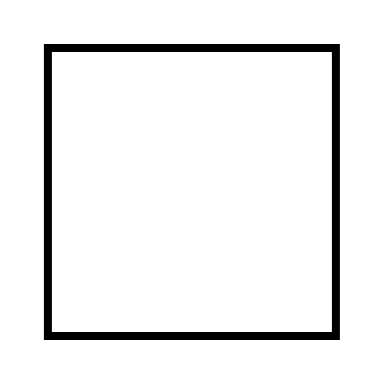 | 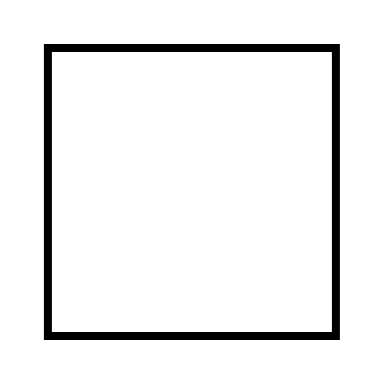 | 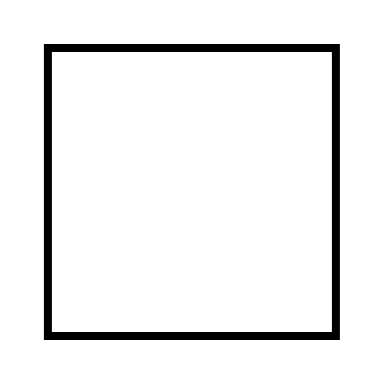 |
| The remote monitoring service display is easy to read | 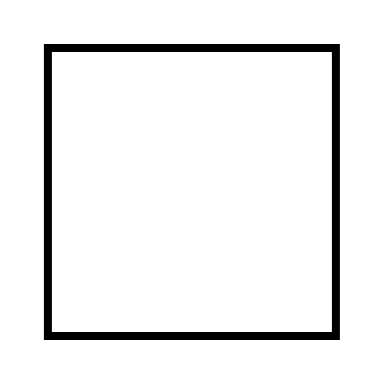 | 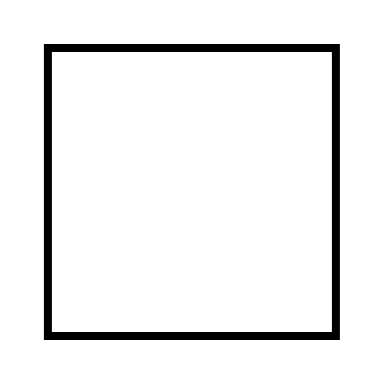 | 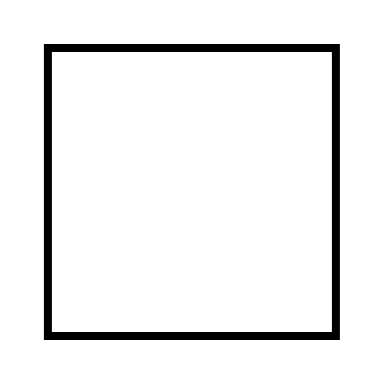 | 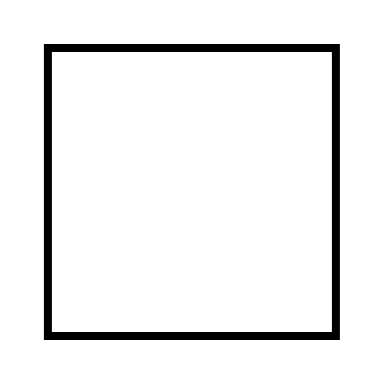 | 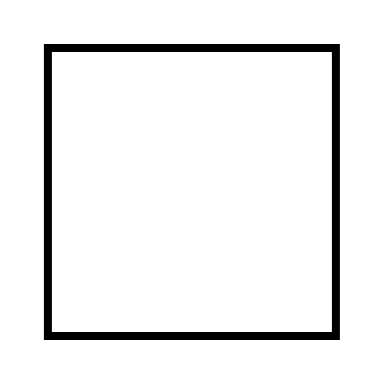 |
| The remote monitoring service is reliable and has few technical problems | 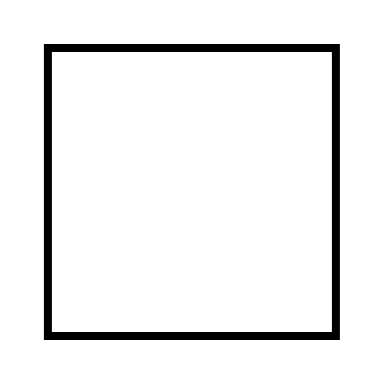 | 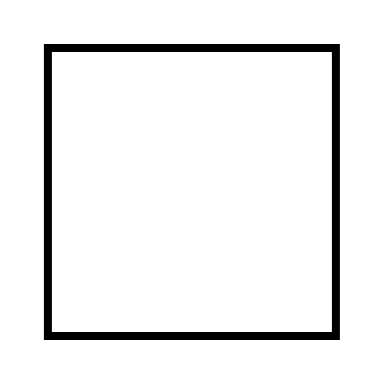 | 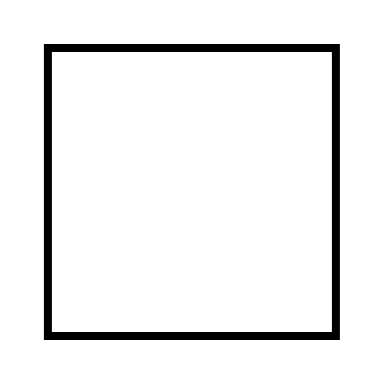 |  |  |
| I received adequate information about how to use the remote monitoring service |  |  |  |  |  |
| If technical problems occur, the staff are quick to respond and fix the problems |  |  |  |  |  |
| The amount of time it takes to complete my remote monitoring questions is acceptable |  |  |  |  |  |

1. Have you ever been advised through the remote monitoring service to contact your rheumatology team? *

- Yes
- No
- Not sure

1. If you were advised to contact your rheumatology team, did you do this? *

- Yes
- No
- Not sure

1. If you did not contact your rheumatology team, please tell us why
2. Please tell us about your experience of contacting your rheumatology team (and using the helpline if this is part of your hospital's service)
3. What should the remote monitoring service start or stop doing?
4. Are there any other comments you would like to make about the remote monitoring service?

**Your confidence in managing your condition**

1. Thinking specifically about your rheumatoid arthritis condition and care, to what extent do you agree or disagree with the following statements: *

|  | Strongly agree | Agree | Neutral | Disagree | Strongly disagree |
| --- | --- | --- | --- | --- | --- |
| I know enough about my health |  |  |  |  |  |
| I can look after my health |  |  |  |  |  |
| I can get the right help if I need it |  |  |  |  |  |
| I am involved in decisions about me |  |  |  |  |  |

**About you**

We would like to understand more about the people using the remote monitoring service, such as age, gender, and ethnicity. We would be grateful if you can answer these questions.

1. How old are you?

- Under 15
- 15 to 20
- 21 to 30
- 31 to 40
- 41 to 50
- 51 to 60
- 61 to 70
- 71 to 80
- 81 to 90
- 91 or over
- Prefer not to say

1. Please specify your gender

- Male
- Female
- Other
- Prefer not to say

1. Which of these best describes what you are doing at present

- Full-time paid work (30 hours or more each week)
- Part-time paid work (under 30 hours each week)
- Furloughed from work (e.g. due to COVID-19)
- Full-time education at school, college or university
- Unemployed
- Permanently sick or disabled
- Fully retired from work
- Looking after the family or home
- Doing something else
- Prefer not to say

1. What is your ethnic group? Choose one option that best describes your ethnic group or background

- White
- Mixed/ multiple ethnic group
- Asian/Asian British
- Black/ African/Caribbean/Black British
- Any other ethnic group
- Prefer not to say

1. Would you be willing to talk to someone from the team that are conducting this survey about your views of the Remote Monitoring service?

- Yes - please fill in your details below
- No

1. Please provide your name and contact details so we are able to contact you. These details will only be used for the purpose of inviting you to tell us more about your experience of using the remote monitoring service, usually via a pre-arranged phone call. *

**Thank you for taking the time to complete this survey. We value your feedback.**

**Supplementary Data S3. Semi-structured interview guides.**

**Supplementary Data S3A. Patient interview guide.**

1. Could you tell me about your age and when you were diagnosed?
2. Could you tell me how you are feeling today in terms of your rheumatoid arthritis?
3. Could I ask you to think about when you first started using the service? Have your views changed?
4. Broadly, how would you describe your experience of the remote monitoring service?
5. What do you understand about how this service works and its purpose?
6. Can you talk me through what happens when you receive the text inviting you to complete the questionnaire?
7. Thinking specifically about the patient questionnaire, how easy do you find it to complete?
8. How does completing the patient questionnaire fit into your routine?
9. Can I ask you about your use of the space to provide free text comments?
10. How do you think the clinical team uses your responses to the patient questionnaire?
11. Do you feel the patient questionnaire gives your clinical team an accurate view of your condition and how you are feeling over time?
12. Do you think the remote monitoring service in general helps you to manage your Rheumatoid Arthritis?
13. What may stop or prevent you from using this service?
14. What may help to overcome these challenges or difficulties?
15. Has COVID-19 changed how you view and experience the remote monitoring service?
16. Do you have any thoughts or suggestions for us that would help the service to work better for you?

**Supplementary Data S3B. Staff interview guide.**

1. What is your (general) view of the remote monitoring service?
2. How easy it is to engage with the different elements of the remote monitoring service?
3. How well do you feel the service fits the needs of clinicians?
4. How is it suitable for all patients and does it reflect their needs?
5. How does remote monitoring compare to face-to-face monitoring?
6. When did you become involved with the remote monitoring service?
7. How did you learn about how to work with the remote monitoring service?
8. What do you know about how this service model has become adopted within the Trust?
9. Talking about supporting factors, what were pre-existing knowledge/skills/resources which initially supported the development of the service?
10. What factors help support the delivery of the remote monitoring service at present within the Trust?
11. Talking about difficulties, were there any challenges or barriers to the introduction of this service?
12. What challenges in your opinion hinder the functioning of remote monitoring service at present?
13. Regarding the implementation of the remote monitoring service, what (strategies) do you think worked well?
14. And what (strategies) do you think did not work well?
15. What factors do you think are important to sustain the remote monitoring service in the long run in the Trust?
16. What factors do you think are important in introducing the remote monitoring service to other Trusts in the future?

**Supplementary Table S1. Extracted data from the rapid evidence synthesis review.**

**Supplementary Table S1A. Study authors, year, country and study type.**

| **Study authors** | **Year** | **Country** | **Study type** |
| --- | --- | --- | --- |
|  |  |  |  |
| Nishigushi et al | 2014 | Japan | Prospective, longitudinal study looking at development and feasibility of a daily assessment system for RA patients using a smartphone app. N = 9 RA patients recorded daily TJC and mHAQ + 15metre walk/day |
| Azevedo et al | 2015 | Portugal | Qualitative study - questionnaire by interview - on use of smartphone apps in RA. |
| Epis et al | 2016 | Italy | Observational prospective study. N = 185 patients randomly assigned to first complete questionnaire by paper/pencil then by tablet or in the opposite order, then asked to indicate preferred version. Completed 1) Visual Analog Scale (VAS) pain; 2) Global Health (GH) score; 3) Patient Global Assessment of Disease Activity (PGA). |
| Salaffi et al | 2016 | Italy | Prospective, longitudinal, case-control study: telemonitoring intensive strategy in early RA vs routine care. Both groups seen every 3 months but intervention group completed RAID in all the interval months. N = 21 intervention, n = 20 control. |
| Riel et al | 2016 | Netherlands | Literature review of existing apps. N=17 articles included |
| Miedany et al | 2016 | UK | Double-blind multi-centre RCT, groups of either monthly ePRO + 3-monthly clinic visit or monthly paper PROM with monthly clinic visit for 6months then every 3 months |
| Yen et al | 2016 | USA | Pilot usability and time motion study. READY mApp - shared platform incorporating data from patients and clinicians. |
| Grainger et al | 2017 | New Zealand | Systematic review of New Zealand iTunes and Googleplay app to identify all RA activity monitoring apps. 19 apps met criteria for inclusion. |
| Walker et al | 2017 | Switzerland | Prospective multi-centre study. Correlation between RAPID-3/4 and DAS28-CRP, CDAI and SDAI. User-friendliness and usability of app rated via system usability scale. |
| Reade et al | 2017 | UK | Mixed-methods study, observational longitudinal, feasibility study [app collecting PROMs + another app linked to GPS collected accelerometer data]. N=20 RA patients for daily data entry, with entry and exit interviews. |
| Mollard and Michaud | 2018 | USA | Pilot mixed-methods study. Quant = 2-3group experimental design, qual = telephone interview for people who did not complete study N = 21 intervention group, n = 15 control group. Qual tele interview n = 12 |
| Najm et al | 2019 | France | Systematic review of existing apps |
| Gandrup and Yazdany | 2019 | USA | Methodology: structured review of the literature and description of the examples from 2 different national electronic collection efforts, 1 in the United States and 1 in Denmark. N = 10 health IT tools. |
| Navarro-Millan et al | 2019 | USA | Qualitative. 7 focus groups with total 31 RA patients |
| Luo et al | 2019 | USA | Systematic review of publicly available apps, directed toward individuals with RA |
| Bhattarai et al | 2020 | Australia | Qualitative study - semi-structured interviews - looking at attitudes/experiences of older people with chronic arthritic pain towards using an app for their pain management. |
| Kuusalo et al | 2020 | Finland | Open RCT comparing text-message enhanced monitoring to routine monitoring of early RA. N = 166. Visits at 0,3,6,12 months. Intervention group received 13 SMSs in week 0-24 with questions on medication issues and PtGA |
| Najm et al | 2020 | France | Mixed-methods. 1) UK focus group and 2) Online international survey across 45 countries |
| Knitza et al | 2020 | Germany | Cross-sectional paper based survey, RA, PsA and AxSpA patients |
| Krusche et al | 2020 | Germany | Cross-sectional digital survey of rheumatologists |
| Grainger and Yazdany | 2020 | New Zealand | Mixed-methods explorative (qualitative, interview) and evaluation (quantitative) study - patients and cinicians re: usability of RAConnect app. |
| Austin et al | 2020 | UK | Qualitative study - semi-structured interview/focus groups - looking at REMORA system collecting daily symptoms over 3 months. |
| Sharp et al | 2020 | UK | Qualitative. Data from recorded clinical consultations (n=17), semi-structured interviews (n=63), 2 focus groups (n=13) – between clinicians, patients, researchers, practitioners. IT specialists and managers. |
| Mollard and Michaud | 2020 | USA | Narrative lit review of RA specific and other self-management apps that could be useful in RA |
| Chahal et al | 2021 | Canada and US | Qualitative study - survey - looking at key factors in RA app adoption. |
| Uhrenholt et al 2021 | 2021 | Denmark | Randomised, crossover, agreement study. To evaluate the agreement of PROMs via DANBIO smartphone app and outpatients touchscreen in patients with RA, PsA or AxSpA. Patients inluded must have completed at least 3 previous PROM questionnaires so they were experienced in this process. |
| Magnol et al | 2021 | France | Observational, cross-sectional, multicentre study. Analysis of the use of eHealth tools (internet, apps) by RA patients in France. |
| Lambrecht et al | 2021 | Germany | Cross-sectional paper based survey of patients with rheumatic disease, after patients tested Rheuma Auszeit for at least 10min using tablets |
| Richter et al | 2021 | Germany | Observational study looking at integrated care platform PICASO - Patients received BP monitor, weighing scale, fitbit, android tablet to collect PROMs . N = 30 patients and 9 clinicians, questionnaires. |
| Muskens et al | 2021 | Netherlands | Observational single-centre study to evaluate use of eHealth platform and SMOC [self management outpatient clinics] in RA in real world setting - assessed whether intervention had a significant effect on health-care utilization and DAS28 compared with the underlying trend. Any RA patient in remission/LDAS could use this. |
| Shaw et al 2021 | 2021 | Switzerland | Observational, cross-sectional survey. To explore the effect of apps measuring PROMs on patient-provider interaction in rheumatic diseases – RA, PsA and AS.  n = 1799/2011 non-app users, 150/2011 app-only users, 162/2011 app+discussion users. |
| White et al | 2021 | UK | Qualitative. 2 semi-structured focus groups lasting 60 mins each, to identify symptoms prioritised by RA patients for inclusion in platform and key requirements for platform to maximise utility N = 9 RA patients. |
| MacIver et al | 2021 | UK | Systematic narrative review looking effectiveness of tele-health interventions to support self-management in RA. N = 7 studies. |
| Nowell et al | 2021 | USA | Prospective longitudinal ancillary study. T understand which PROMs patients with RMDs consider most important to track for disease management. ArthritisPower registry patients with AS, RA, PsA, SLE, FM, OA and OP. Analysed PROMs with high weighted summary scores and rank-order prioritisation. |
| Lee et al | 2021 | USA | 6 month RCT of care coordination alone or with app intervention [daily ePROMs]. N = 191 RA patients. |
| Seppen et al | 2020-a | Netherlands | 2 mixed-methods pilot studies, feasibility study. Two 1-month pilot studies n = 42 and 27 RA patients, where they completed a weekly questionnaire for 4 weeks via app |
| Seppen et al | 2020-b | Netherlands | Systematic scoping review looking at asynchronous mHealth interventions in RA. Found 4 types of mHealth interventions used - SMS eminders (n=3), web apps (n=5), martphone app (n=1), activity tracking (n=1). |

**Supplementary Table S1B. Patient acceptability.**

| **Study authors** | **Year** | **Country** | **Patient acceptability** |
| --- | --- | --- | --- |
|  |  |  | ***Usability *Patient knowledge *Motivation/preferences** |
| Nishigushi et al | 2014 | Japan | Post-observation period questionnaire: could you record your body condition and your life every day? [no problem: n=8/9]. Did you feel the feedbacks of the system suitable to your body condition? [very suitable: n=7/9]. Were the feedbacks helpful for you to live your life? [helpful: n=6/9]. Did you feel nervous or confident to share information (about gait and body condition) with doctors? [confident: n=7/9] |
| Azevedo et al | 2015 | Portugal | N = 100 RA patients.  86% said it would be useful to develop a RA self-management app, and of these 83% said they would use it [these were younger, had access to a smartphone and using sms/email/internet].  82% were willing to pay for it.  64% wanted data automaticaly sent to rheumatologist |
| Epis et al | 2016 | Italy | For all the evaluated items, the intrarater degree of agreement between 2 approaches was found to be excellent (intraclass correlation coefficient>0.75, P<.001). No significant differences found between the two approaches, including for which version was preferred by patients (p > 0.99) |
| Salaffi et al | 2016 | Italy | Patient satisfaction with telemonitoring system was high and 90.5% said that they would continue to use it in the future |
| Riel et al | 2016 | Netherlands | N=1 study found manual disability in patients with RA is not an obstacle for using mobile apps - [n=15] patients agreed app is easy to use, intuitive and mobile VAS is at least as easy to complete as in paper form. |
| Yen et al | 2016 | USA | Usability evaluation (n = 33 patients, 15 physicians) - problems found in navigation, data entry and pain assessment [not intuitive], documentation [physicians documented diagnoses but missed task steps in changing medication] and instruction along with error messages.  TMS: overall patients spent more time on READY than on paper, but overall it did not delay workflow. 75.8% patients reported they liked READY mApp |
| Walker et al | 2017 | Switzerland | The System Usability Scale indicated a median score of 85 (IQR: 73-93) at the follow-up visit, suggesting that patients rated the WebApp as very positive in terms of ease of use. Patients rated the benefit of the WebApp on the patient-physician communication with a median score of 74 (IQR: 43-93), on the 100-mm visual analog scale. |
| Mollard and Michaud | 2018 | USA | P-SEMS in intervention group had a statistically significant improvement to control (2.8 vs -1.66, p = 0.04) |
| Najm et al | 2019 | France | 70% of apps directly evaluated through a satisfaction scale and/or through an open/closed questionnaire (52%) - these were generally positive. |
| Gandrup and Yazdany | 2019 | USA | From the patient’s point of view, minimizing data collection burden is essential and fatigability of use is important. Technology, such as smartphone apps, enables patients to generate important data outside of the hospital setting as often as needed and share it with their providers to expand the depth, breadth, and continuity of information available |
| Navarro-Millan et al | 2019 | USA | Looking at RA perspectives on PROM collection, 3 themes - provider communication [keen to communicate soon after symptoms but less keen if doing well]; information seeking about RA [interested in learning from others with RA but most prefer to learn from provider and very few aware of PROM colelction that can track symptoms and share with clinician]; social and peer support [from health care team, family, others with RA].  Barriers to electronic data collection at home are fatigue, pain, limited capability due to hand/wrist symptoms. |
| Bhattarai et al | 2020 | Australia | N = 16 4 themes - apps are valuable for self-management but potential for harm, a pain management app needs to strictly align with the user's needs, clinician involvement is crucial, the app must be designed with the user in mind. Older people found being able to diarize symptoms added value, as did being given relevant exercise instructions. Some concernes expressed - could lead to them overfocusing on pain |
| Kuusalo et al | 2020 | Finland | 100% of patients would recommend SMS monitoring for other RA patients, but 25% found the self-assessment of disease activity using PtGA somewhat difficult/difficult. |
| Najm et al | 2020 | France | N = 6 in focus group {5 RA, 1 myositis] and N = 394 survey 52.3% were aware of the existence of apps to support them in self-managing RMDs – of these, 42% were currently using a self-management app, a third of them on a weekly basis.  The majority of patients were interested in an app that helped with self-monitoring of health parameters (259/346, 74.9%), disease activity (221/346, 63.9%), communication with their health care providers (221/346, 57.8%), and information about their disease (200/346, 53.5%). |
| Knitza et al | 2020 | Germany | N = 193 68.4% believed medical apps could be beneficial for their own health, 66.8% would regularly enter data into an app, 75.6% would welcome app recommendations from national rheumatology societies; preferred duration for data entry was no more than 15 mins [57%] and preferred frequency was weekly [30.6%]; most desired app feature was medication information 77.7%. The mean eHealth literacy was low (26.3/40) and was positively correlated with younger age, app use, belief in benefit of using medical apps, and current internet use to obtain health information |
| Grainger and Yazdany | 2020 | New Zealand | N = 9 RA patients Themes - variable acceptance/readiness with some enthusiastic and some concerned about technical skills and reduction in clinician contact; app use reducing barriers to access to care; the app did not capture some pain and function aspects of lived experience; allocation of clinics by need  N=16 RA patients Usability: SUS own device for 1month = 79.5 |
| Austin et al | 2020 | UK | Patients identified 3 themes - RA as an 'invisible disease' due to fleeting symptoms, ePROM system provided bigger picture of RA and it enabled patient-centred consultations |
| Sharp et al | 2020 | UK | Explores challenges of achieving effective collaboration in the development/use of a novel healthcare innovation drawing upon the concept of boundary object: Boundary between technical innovation and clinical practice – needs to fulfil patient and clinician needs, Boundary between disease management at home and that in the clinic [Patients felt graphs removed the burden of communicating their disease to the clinician], Boundary between the implementation of the innovation from a single research clinic to wider scale-up |
| Chahal et al | 2021 | Canada and US | N = 30, 73% male.  "track symptoms" and "schedule appointments/ receive reminders" rated highest amonst patient preferences.  Paying for an app - 33% willing, 30% not willing, 37% unsure |
| Uhrenholt et al 2021 | 2021 | Denmark | HAQ-DI scores were equivalent for the two device types, with a difference of −0.007 (95% CI −0.043 to 0.030). Subgroup analyses showed no significant difference in HAQ-DI score between genders, diagnosis (RA vs PsA/axSpA), younger half versus older half, or participants > 65 years old versus younger.  *All other PROMs obtained with the two device types were equivalent (VAS pain/fatigue/global health), except for the BASDAI - 95% CI for the difference in BASDAI (−5.4 to 2.4) exceeded the prespecified equivalence margin of ± 5.0.  ** 47/68 (78.3%) preferred PROM data entry through DANBIO app to outpatient touchscreen |
| Lambrecht et al | 2021 | Germany | N = 126 RA, PsA and AxSpA patients 70.% believe medical apps useful for health and 65.9% willing to use them in the future, preferably for a max time of 5-15 mins [34.1%]; 74.6% interested in an app including physical exercises and stress reduction activities |
| Richter et al | 2021 | Germany | N=12/29, 41% of patients felt better understood by their rheumatologist about their complaints, and n=22/24, 92% reported an easier communication with treating rheumatologist. |
| Shaw et al 2021 | 2021 | Switzerland | Respondents in the app+discussion group were more satisfied with the apps – 92% of app+discussion vs 72% of app-only group found the apps easy to use (p<0.001), to understand (95% vs 83%, p=0.004) and were more likely to recommend the app to others (72% vs 41%, p<0.001) |
| White et al | 2021 | UK | 4 themes - Key symptoms are pain, mobility mood, Key outcomes are social life, work life, parenting; ways in which patients manage RA symptoms [education on medication, credible info on complimentary therapies eg diet and exercise]; views on current RA healthcare system - Importance of continuity of care, patient-centred care and the impact of symptom fluctuation on appointment effectiveness, patients agreed being able to log flares to then report at visit is beneficial; views on remote measurement in RA - ability to track symptom changes in pain/mobility within and in-between days, by body part, and track potential triggers eg sleep, exercise and ability to review what is tracked over time |
| Nowell et al | 2021 | USA | N = 253 completed initial selection of PROMs at baseline.  PROMIS Fatigue had the highest weighted summary score overall (54.8), followed by PROMIS Physical Function (41.3), PROMIS Pain Intensity (40.7), PROMIS Pain Interference (39.5), Duration of Morning Joint Stiffness (29.6), and PROMIS Sleep Disturbance (28.1). After calculating the mean (SD) of participants’ rankings for each PRO overall, the same rank-order prioritization of symptoms was observed for the top six |
| Lee et al | 2021 | USA | Of the n = 67 intervention group who completed the exit survey, 90% rated their likelihood of recommending the app as at least 7 of 10. 87% agreed the app helped track disease activity, 54% agreed that information from app was incorporated into clinic visits; 58% thought the combination of app and care coordinator was the most helpful component |

**Supplementary Table S1C. Clinician acceptability.**

| **Study authors** | **Year** | **Country** | **Clinician acceptability** |
| --- | --- | --- | --- |
| Walker et al | 2017 | Switzerland | Physicians rated the benefit of the WebApp on the patient-physician communication with a median score of 50 (IQR 18-60), on the 100-mm visual analog scale. |
| Krusche et al | 2020 | Germany | N = 119 clinicians 89.9% used any form of PRO daily; however, 10.1% indicated that they did not collect PROs at all. Of the rheumatologists using PROs, 23.5% collected PROs electronically at each patient appointment and 4.2% collected PROs electronically before patient contact. |
| Grainger and Yazdany | 2020 | New Zealand | N = 7 clinicians Clinicians concerned technical abilities needed could exceed those of patients and clinicians; felt it was useful for self-management but concern app use could increase RA-related anxiety in patients seeing self-reported measures quantified |
| Austin et al | 2020 | UK | Themes - provides bigger picture of RA and enables shared decision making and patient-centred consultations |
| Richter et al | 2021 | Germany | N=21/28, 75% of clinicians were satisfied with the clinician dashboard. Overall benefit of additional health data for the RA treatment in clinic visits was rated Likert 2.3. |
| Lee et al | 2021 | USA | Of the 11 physicians who completed the exit survey, 73% agreed/strongly agreed that they wanted to continue offering the app to patients. |

**Supplementary Table S1D. Patient engagement.**

| **Study authors** | **Year** | **Country** | **Patient engagement** |
| --- | --- | --- | --- |
| Reade et al | 2017 | UK | Successful engagement reasons – simple graphical user interface, automated reminders, visualisation of data, interest and perceived value of research.  Barriers to ongoing engagement – reduced phone battery life due to app, perceived lack in technical skills. |
| Mollard and Michaud | 2018 | USA | Qualitative themes about barriers to app use from interviews with dropouts - 1. Frustration with technology, 2. RA made the app difficult to use and 3. Satisfaction with current self-management system. |
| Najm et al | 2020 | France | Reported app usage – (42/67, 62.7%) for disease management, (28/67, 41.8%) for coping with arthritis symptoms and consequences, (24/67, 35.8%) for medication intake monitoring. Those who stopped using the app cited that they did not find them helpful (24/37, 64.9%), saw no benefit for their health (16/37, 43.2%), found the device too time-consuming (15/37, 40%), got bored of using the app (11/37, 29.7%), or they did not like the design or user interface (10/37, 27%). |
| Mollard and Michaud | 2020 | USA | Barriers to mobile app use include patient preference, resistance to technology adoption, quality of app, hand disability and concerns about security.  Apps that connect users to rheumatology providers – allows better overall picture of the individual’s RA clinical course; patients likely to submit self-monitoring data with the knowledge it was sent to their rheumatologist/electronic health record. Patient collected data in graphical format can reveal patterns of disease activity that may not be noted without the app. |
| Magnol et al | 2021 | France | N = 575 patients 82.2% have access to eHealth tools; 28.7% used them for RA and of these, 100% used the eHealth tool to obtain information about RA; 66.4% used it to monitor their RA. Univariate analysis showed that age, education level, employment status, treatment, comorbidities, membership of a patient association, and patient education program were associated with eHealth use for rheumatoid arthritis. Multivariate analysis - Patient member ship of a patient association was independently associated with use of eHealth tools for RA (OR 5.8, 95% CI 3.0-11.2, p<0.001), and High level of comorbidity (OR 0.7, 95% CI 0.6-0.8, p<0.001) and use of biologic DMARDs (OR 0.6, 95% CI 0.4-1.0, p<0.041) were associated with a lower use of eHealth tools. |
| Muskens et al | 2021 | Netherlands | On average, patients using the eHealth platform and participating in SMOC tended to be younger, more highly educated and have better health outcomes, use more medication, consume more alcohol and be more likely to have stopped smoking. |
| Shaw et al 2021 | 2021 | Switzerland | App users were younger than non-app users (47 vs 51 years, p<0.001) and had a higher proportion with LDA at baseline (58-64% vs 51%, p<0.01).  In adjusted analyses, compared with non-app users, the app+discussion group was more likely to be satisfied with shared decision making (OR 1.66, 95% CI 1.14-2.42) and with physician disease tracking (OR 2.0, 95% CI 1.3-3.09), whereas the app-only group had similar levels of satisfaction with these outcomes compared with non-app users. |
| Seppen et al | 2020-a | Netherlands | App usage declined over time in both pilot studies: from 100% and 78% in week 1 to 61% and 37% in week 4 in pilot 1 and 2 respectively. 81% (25/31) said they would like to skip hospital visits if the self-monitored disease activity is low.  Promoters for usage identified: ‘experiencing more grip on their disease’, ‘improved communication with their physician’ and pain i.e. more pain promoted and less pain discouraged app usage.  Barriers for usage identified: technical problems, internal resistance (respondent fatigue, app reminded them of their disease, and a lack of symptoms |

**Supplementary Table S1E. Clinician engagement.**

| **Study authors** | **Year** | **Country** | **Clinician engagement** |
| --- | --- | --- | --- |
| Krusche et al | 2020 | Germany | Question "why are PROs not used?" answered by n = 68.  34% stated that they did not know a specific software, 12% said that the introduction of a software was too complicated, and 12% thought the software was too expensive. 18% of nonimplementers reported that using ePROs was too time-consuming, 16% reported that patients preferred paper-based questionnaires, and 32% stated “other reasons”. |

**Supplementary Table S1F. Feasibility.**

| **Study authors** | **Year** | **Country** | **Feasibility** |
| --- | --- | --- | --- |
| Walker et al | 2017 | Switzerland | At baseline there was a moderate to strong correlation between RAPID3 and DAS28 (r = 0.63), CDAI (r = 0.65) and SDAI (r = 0.61) scores. Similar or stronger correlations were seen at the 3-month follow-up visit (DAS28 r =0.66, CDAI r=0.71 and SDAI r = 0.61). |
| Reade et al | 2017 | UK | 30% withdrew [technical challenges, health reasons]; mean completion rate for data entry was 68% over 60 days, |

**Supplementary Table S1G. Clinical impact.**

| **Study authors** | **Year** | **Country** | **Clinical impact** |
| --- | --- | --- | --- |
| Salaffi et al | 2016 | Italy | Time to achieve CDAI remission shorted in intervention group [median of 20 weeks versus a median over 36 weeks (P <0.001)] and higher percentage in intervention group achieved CDAI remission (38.1 % vs 25 % at year 1, P <0.01) Intervention group had statisticaly significant lower total radiographic progression than control |
| Miedany et al | 2016 | UK | No significant difference between DAS28 and RAPID-3 in both groups at 3, 6 and 12 months of management  Patient adherence to immunosuppression was higher in the active group vs control group (89.6% vs 70.5%, p<0.01)  Fewer patients in the active group stopped medication due to intolerance vs control group (5.7% vs 19%, p<0.01) Control group patients had a higher number of visits due to flare needing early assessment during study period (37.1% vs 21.7% active group, p<0.01) |
| Kuusalo et al | 2020 | Finland | No statistically significant difference in remission rates or mean DAS28 at 6 or 12 months between groups.  During intervention, the use of healthcare resources increased in intervention group: The mean ± SD number of nurses’ telephone contacts was 3.32 ± 2.93 in the intervention group and 2.0 ± 2.55 in the control group (P = 0.008). |
| Mollard and Michaud | 2020 | USA | n =1 Study looking at PROMs with patient connected to a population manager who monitors for increased disease activity or communicates at set intervals - patients did not have increased satisfaction or reduced disease activity, but app adherence was strong and baseline satisfaction was already high. |
| Muskens et al | 2021 | Netherlands | The mean DAS28 (3.19) decreased significantly (relative to the pre-interruption trend) by 0.056 per quarter (95% CI: -0.086, -0.025, P=0.001).  The mean DAS28 (2.94) of those participating in SMOC, the quarterly post-interruption trend of the DAS28 stayed approximately the same (p=0.962)  The mean number of quarterly outpatient clinic visits per patient decreased by 0.027 per quarter (95% CI: -0.045, -0.08, P=0.007). |
| MacIver et al | 2021 | UK | No significant differences at 6 months for disease activity, pain or fatigue [n=2]. Web-based self-monitoring app showed higher percentage achieved statistically significant remission in intervention group with shorter time to CDAI remission [n=1] (38.1% vs 25% at 12 months p = < 0.01). Telebased self-management post-discharge: n=1 found no difference in disease activity at 12 and 24 weeks, and n=1 found significant improvements in intervention group HAQ-DI at week 8 (1.17 vs 1.46 p = 0.01). Medication adherence [n=2] higher in self-management website and tele self-management education groups. In healthcare utilisation, n=1 showed no difference with SMS self-monitoring; but n=1 showed significant decrease in visits if social support section (p=0.02) and gaming features (p=0.03) used in web-based self-management |
| Lee et al | 2021 | USA | The mobile app designed to collect ePRO data on RA symptoms did not significantly improve patient satisfaction or disease activity compared to care coordination alone.  No statistically significant group differences in the medians of TSQM, PEPPI, or CDAI scores at 6 months were detected. |
| Seppen et al | 2020-b | Netherlands | SMS messaging - n=1 RCT showed – weekly text messages for medication adherence in MTX in RA, showed increase in medication adherence [9-item CQR] compared to control and group receiving one pharmacists counselling session.  * Allam et al – patients with access to social support sections of app decreased health care utilisation and medication overuse; those with access to gamification +/- social support increased physical activity and decreased healthcare utilisation  Shigaki et al online platform with educational modules encouraging positive coping strategies – platform improved self-efficacy and QOL in intervention group but no significant improvements were seen in terms of health status or pain in intervention group Salaffi et al – telemonitoring intensive strategy (TIS) group collecting PROms vs conventional strategy group (CS). More patients in TIS group achieved CDAI remission and achieved remission more rapidly vs CS group.  * Smartphone app - One RCT used optical imaging and self-management with features to monitor symptoms - after 6-month app use, significant improvement seen in PROMIS and P-SEMS |

**Supplementary Table S1H. Available applications (apps).**

| **Study authors** | **Year** | **Country** | **Available apps** |
| --- | --- | --- | --- |
| Grainger et al | 2017 | New Zealand | This review found a lack of high-quality apps for longitudinal assessment of RA disease activity. Current apps fall into two categories: simple calculators primarily for rheumatologists and data tracking tools for people with RA. The latter  do not uniformly collect data using validated instruments or composite disease activity measures |
| Najm et al | 2019 | France | 81% of apps were linked to a specific rheumatic disease, 34% were for RA. 18% were designed for multiple diseases or for general population but used by patients with RMDs.  68% of apps designed for self-monitoring and collection of specific outcome measures including PROMs.  77% allowed self-visualisation of health data as a trend.  21% aimed to promote physical activity through daily reminders and education.  • 9% of studies did not describe development process at all, and 15% stated patients included in development process. 40% of studies involved health professionals/physicians in development or evaluation phase. |
| Luo et al | 2019 | USA | App purpose split into 3 categories: exclusively symptom tracking 50%, exclusively patient education 25%, cmbination of both 25%.  Of the 15 apps that enabled symptom tracking, 87% had features that generated graphs from self-reported data and 67% linked to a website for further information. 60% of apps used a visual analog scale for symptom tracking and 53% apps provided tools to track disability/QoL. 26% had ability to input TJC/SJC through joint diagram and 26% enabled users to input lab results |

**Supplementary Figure S3. Rheumatoid Arthritis Impact of Disease (RAID) score completion rates in relation to the number of requests sent.**

**Supplementary Table S2. Average Rheumatoid Arthritis Impact of Disease (RAID) score completion rates according to cumulative time onboard the remote monitoring (RM) service.**

**Supplementary Table S3. Staff interview results showing categories, codes, key findings and example quotes mapped across the Exploration, Preparation, Implementation, Sustainment (EPIS) framework.** DPC (Digital Pathway Coordinator); RM (Remote monitoring); PROM (Patient Reported Outcome Measure).

| **Phase** | **Construct** | **Category** | **Code** | **Key findings** | **Example Quotes** | |
| --- | --- | --- | --- | --- | --- | --- |
|  |  |  |  |  | **Facilitators** | **Barriers** |
| **Exploration** | **Inner context** | **Leadership** | **Involvement** | Varying levels of involvement across sites from 2017 to 2020. | *"since the early part of remote monitoring I think I’ve been somebody who’s been consulted from the developer’s point of view. I had the opportunity to see the progress: […] So I’ve been privy to that information before and been able to voice any concerns that I’ve had". [Staff 11]* | *"I was also, initially, just involved purely as a clinician just recruiting patients" [Staff 2]* |
|  |  |  | **Needs assessment** | Variable awareness of and involvement in needs assessment activities across sites, which were felt to focus on patient needs. | *"I think it’s come from a design idea that's got a bit of legs that then went into a design space, and then went into a lot of patient engagement work to really get the messaging right, the visuals right." [Staff 15]* | *"So I wasn’t aware what was done, but I was aware it was done" [Staff 2]* |
| **Preparation** | **Innovation factors** | **Innovation characteristics** | **Development team** | Digital product development team were supportive and engaged. | *"They were really helpful and they listened to us. We had meetings with them regularly about what was being done. What changes they were making. Ideas that we were able to share with them" [Staff 8]* | *N/A* |
|  |  |  | **Digital platform** | Many felt the platform was well-designed and user-friendly, but one individual felt it was not ready for clinician engagement. | *“I sat in clinic; I sat over the shoulder of a number of clinicians getting them to have a go at using it. And it was clear that it was a simply designed process” [Staff 14]* | *"I think the problem was when before we were doing it the system wasn’t really ready for that. It wasn’t ready for them to be using it. […] [that] held me back from showing clinicians how to use it…" [Staff 8]* |
|  |  |  | **Administrative support** | The DPC has a central role in managing the RM service, which makes it easy to use. This ‘human factor’ also enables effective triaging of patient responses. | *"the service has been entirely kind of concierge by [digital pathway coordinator], … you email [digital pathway coordinator], … she put the patient on, and she'll let you know if that patient triggers a signal through the monitoring" [Staff 4]* | *N/A* |
|  |  |  | **Service design** | The RM service was well-designed for both staff and patients. Existence of a previous database laid important groundwork. | *"[Project Lead] wanted us to get into the habit from the beginning of trying to be a bit personal with patients, not inappropriately but to make the message to the patient personal and to say things like, we're glad... we're really pleased to hear that you're doing so well or we're sorry..." [Staff 1]* | *N/A* |
|  |  | **Fit to clinicians** | **Use alongside face-to-face appointments** | Although the RM service should not replace face-to-face appointments, many felt it could be a useful complement and creates the potential to allocate and use face-to-face appointments more effectively. However, some questioned the necessity and value of the service. | “*I can space out patients’ appointments less frequently because I know that they can contact us and they contact the service and have advice if they need to, if they flare. That gives me confidence to stretch the appointments to more lengthy intervals". [Staff 9]* | *"The patients that is aimed at is the stable patients. And they all have a safety net appointment and they all have a helpline. And if they're stable and they're copped on enough to fill in prompts, their copped on enough to ring the helpline. And they do." [Staff 16]* |
|  |  |  | **Potential to support clinical decision making** | Potential to support clinical decision making, as regular capture of PROM scores can provide a more objective representation of a patient's disease activity over time, but limitations exist. | *“It’s difficult for me to sum up the course of the year’s events in a 20 minute consultation. Things happen, and they may forget the disease control, they might forget flares, and so we won’t get a real true representation of how they are” [Staff 11]* | *" the subdomains of the scale will give some indication as to what the problem is, but all it will do is give you, will indicate need or risk that a patient needs intervention, but it doesn't it doesn't have the granularity to tell you exactly what's required" [Staff 4]* |
|  |  |  | **Unmet expectations** | Expectations of developing a ‘database’ for all patients not delivered. | *N/A* | *"what we were promised, what we thought it was going to be and what the department wanted is not what’s been created essentially." [Staff 13]* |
|  |  | **Fit to patients** | **Patient characteristics** | Suitable for most patients but not all, including those with more active disease, a secondary pain diagnosis, those with poor communication skills and who are unable or unwilling to use the internet. Some sociodemographic barriers may be more prevalent in certain contexts. | *"I think it’s suitable for the majority of patients, not all" [Staff 10]* | *“This area is very socioeconomically deprived. And there's a lot of people with very chaotic lives. And a lot of the difficulties that we have to manage is more lifestyle, socio economic, type of stuff, because it's all rolled in with, the patient is that whole being, aren't they, it's not just they've got rheumatoid. And so a lot of the barriers to the engagement with the patient is their background, their socio economic, their household, their lifestyle" [Staff 16]*  *“I think that works for people who have good self-advocacy and are not too emotionally distressed" [Staff 12]* |
|  |  |  | **Fit into day-to-day routine** | RM service is quick, easy and non-intrusive for patients, versus burdensome, anxiety-inducing and an unwelcome reminder of disease. | *"I was told many, many times by patients, they do not wish to be reminded they are ill when they are well, so it's giving the patients an opportunity to get on with their lives, but when it goes wrong, they've got a quick way to get back into the system." [Staff 14]* | *"On the flip side, I suspect some [inaudible 07:28] of patients will be quite happy to forget that they have a rheumatological disease for 24 months until it's time for the appointment, and yet they're being reminded by it every month or two months" [Staff 15]* |
|  |  |  | **Connection and safety** | An alternative and potentially more effective line of communication for patients, which can offer a feeling of connection, safety and improved access to care. But worry that this could replace face-to-face care. | *"especially for patients that aren’t reviewed very often, that they still feel, like, somebody is there and that they’re still being monitored" [Staff 8]* | *“their main worry is that this doesn’t replace their routine clinical care, and they all said, ‘I hope still get to see you.’ And I think there’s this belief that patients don’t want to come and see their doctors, […] the message I hear from patients is, ‘I want to see somebody.’” [Staff 11]* |
|  |  |  | **Promoting self-management or dependence** | Offering patients more autonomy and empowerment, which can support self-management, versus encouraging overreliance on clinical services. | *"Patients feel more empowered. … When they see their data, they can look for their own trends, they can also go, Ah that was a bad month because…" [Staff 14]* | *"I think it can lead to over reporting on stuff that's not so relevant. And over anxiety on stuff that doesn't need addressing and over reliance, you know, we're trying to promote self-management."*  *[Staff 16]* |
|  |  | **Fit to system** | | Blending face-to-face and remote working is important for future service provision. | *"I think that a blended approach of a mixture of face-to-face and remote clinical assessments for patients with chronic inflammatory disease is what’s going to happen for the future. … to make things more efficient and yet not to lose kind of detail as to what’s happening with patients..." [Staff 5]* | *N/A* |
|  |  | **Adaptations** | | Considerations made to adapt service to suit local needs. | *"We explained a little bit more about the responsibility locally for the nurses to triage the information. And we encouraged them to develop their own standard operating procedure of how they wanted to do that, because we appreciated that every hospital had a slightly different workforce structure" [Staff 14]* | *N/A* |
|  | **Inner context** | **Leadership** | **Leadership characteristics** | Passionate and engaged multidisciplinary leadership team. | *"we’ve had a Team of people working both from [Digital Product Developer] and administrative wise and various clinicians who have involved along the journey" [Staff 9]* | *N/A* |
|  |  |  | **Teamwork and collaboration** | Desire for shared understanding and collaboration, but concerns and feedback not always taken on board. | *"I think the initial consultation, reaching out, getting people to get involved, say what they thought. Say what they liked or they didn’t like. So getting people together to talk about the idea I think was key." [Staff 5]* | *"...the number one barrier was asking us for input and how to develop it, and not responding to it." [Staff 1]* |
|  |  |  | **Communication** | Communications were varied, focussed on the patient perspective, and served as reminders, to report progress and elicit feedback. | *"we did get, you know, various emails and demonstrations about how to do it and little posters went around that we could up in clinics to say this is how you recruit people.” [Staff 5]* | *N/A* |
|  |  |  | **Advocates** | The varied success of appointed champions was bolstered by the emergence of unexpected advocates. | *"having some champions for the service at Guy’s was helpful. People who, like [Clinical Lead] very enthusiastic, passionate about how the system could help and could work I think was very important really." [Staff 5]* | *"But of course, very typically, it is the nurses who are going to be really close to this project, this service, the patients who are using it. ... there was there was more dialogue through the email with nurses than there were with some of the lead clinicians." [Staff 14]* |
|  |  | **Clinician factors** | **Clinician characteristics** | Facilitators include clinical and digital acumen, familiarity with RM technology, but clinical experience may dictate levels of engagement. | *"Well nothing really except clinical acumen. I mean, it's just simply understanding the disease and the rationale for it.” [Staff 4]* | *"I think that we use it in different ways. But having spoken to a couple of colleagues who are a bit like me they say that they still need to see the patient anyway at an interval. Others say, ‘well they’re in remission, they’ll stay in remission it’s fine. We don’t need to see them.’ I think that those who are in that camp tend to be the more senior consultants who have been doing this longer and are probably more comfortable with taking on that risk. I think the people who are less likely to do that are the younger consultants who say, ‘we can use it but it’s not going to replace what we do.’” [Staff 11]* |
|  |  |  | **Teamwork and collaboration** | Local teamworking efforts seen as a facilitator. | *“We agreed a process which was that it was going to be done via our specialist nurses. So there was quite an attempt to make sure that everyone knew how to do it." [Staff 5]* | *N/A* |
|  |  |  | **Attitude towards innovation** | Fear of increased workload, loss of ‘control’ over patients, and lack of ‘confidence’ in the RM service. | *N/A* | *"doctors will worry that they are going to be given extra work because what if every time a score goes up you are contacted about all of your patients with rheumatoid arthritis and you have 100s of them, and you’re already over worked, what are you going to do then?” [Staff 2]* |
|  |  |  | **Readiness for change** | Potential lack of readiness for change. | *N/A* | *“some clinicians probably just won’t engage with any new measures in a department that they’ve been in for 40 years. They’re just used to a way of doing it, and they might never engage"[Staff 2]* |
|  | **Outer context** | **Leadership ties** | | Clinical Lead liaised with commissioners and senior boards. | *“[clinical lead] is, he is part of the Traditional Transformation Board. So I think that’s really useful because you want to know that you’re in keeping with the NHS long term plan and the trust strategy for digital change, which is very, very important for the trust" [Staff 2]* | *N/A* |
|  |  | **Inter-organisational environment** | | Similar platforms may compete for clinicians’ attention. | *N/A* | *"I think one of the reasons that’s a bit difficult is the British Society of Rheumatology around the same time, so early last year, released the BSRE proms platform where any … and it’s not just rheumatoid arthritis but even vasculitis, other conditions, patients can be put onto there." [Staff 2]* |
|  |  | **Climate** | | The coronavirus pandemic created favourable implementation conditions, as the RM service helped meet evolving needs. However, these advantages were offset by barriers introduced by the pandemic. | *"I think the timing with the pandemic coming made it more valuable...Just because we had less contact with patients." [Staff 10]* | *"it was really hard getting engagement from the other sites. So even with the clinicians because we weren’t seeing them. We weren’t face-to-face of people. So it was really hard to, kind of, understand what was holding them back from using it" [Staff 8]* |
|  | **Bridging factors** | | | NHS Innovation Team provided support and funding. | *"I think that [NHS Innovation Team] was helpful […] it’s actually NHS getting involved and trying to say, yes, we think this is a good idea and let’s put some resource into it." [Staff 5]* | *N/A* |
| **Implementation** | **Engagement** | | | Staff engagement was variable over time and greatest at the pilot site. Recent increases attributed to innovation adaptations. | *"But the uptake with the service has been pretty good, some clinicians are less slower than others, as always the way you get the early adopters, and sort of stragglers, the other end, but everyone is contributing to it". [Staff 4]* | *"there’s very limited uptake across the board” [Staff 10]* |
|  | **Innovation factors** | **Innovation characteristics** | **Digital platform** | Several limitations mentioned, including lack of automatic start-up and visual prompts, log-in difficulties and lack of interoperability with existing programmes. | *N/A* | *"I didn’t want to have to open up yet another programme. You know, you have like ten things already open on your desktop and then you need to open one more and if it logs off you’re constantly trying to keep them all. You know, always trying to put your password in to stop everything logging off." [Staff 6]* |
|  |  |  | **Administrative support** | Good admin support, for example with recruitment, monitoring and clinical escalation, makes the service straightforward for clinicians to use. | *"my concern initially when it all rolled out was we were really overwhelmed with questions on what to do with these patients who have written back on this free text box that they’ve got problems, but it doesn’t seem to be too bad, and I do think they bat away the majority of the queries so that’s good and very helpful" [Staff 13]* | *N/A* |
|  |  |  | **Training** | Staff spoke positively about training, with some informally adopting a ‘train-the-trainer’ approach. However, the requirement for training to permit log-in access also acted as a ‘limiting factor’ and discouraged engagement of the innovation. | *"I’ve not had training, although it’s a very intuitive service, and one of my colleagues who have used it, then showed me how to use it." [Staff 10]* | *"it was more that for some patients I felt that if they’d been filling in the data and then when they came to see me I didn’t have their data that was going to be a problem" [Staff 12]* |
|  |  | **Fit to clinicians** | | Not being able to identify which patients are onboard the service acted as a barrier. | *N/A* | *“At the moment, you only expect, you know, a fraction of patients to be on it. And maybe you do a whole clinic and have nobody on it. So it's difficult to remember who is on it and who isn't." [Staff 4]* |
|  |  | **Adaptations** | | Adaptations to address staff needs received mixed reviews. These included increasing the availability of training, making the platform more user-friendly, relaxing patient eligibility criteria and refining triaging processes. Shifting the responsibility of patient management towards clinicians was particularly controversial. | *"… now I see it as a place, not just to put the remission patients, but also if we can put other people there and start collecting PROMs from there and showing them and using it like it is a database to begin with then it becomes a much more relevant part of clinic life." [Staff 6]* | *"Currently, it's relying on clinicians to put and transfer the data in. And that, on top of a very busy clinic, filling up lots of other forms, you know, ploughing through backlog, struggling with technology, etc., it's a mammoth task. So I think that's challenging" [Staff 15]* |
|  | **Inner context** | **Leadership** | **Leadership characteristics** | Clinician involvement brought new insights, but lack of clear leadership seen as a barrier. | *"we’ve learnt different ways of how we can use it, so things that I wouldn’t have known would have been useful for a clinician now that [Clinical Lead] and [Clinical Fellow] are on it and they can see that there’s ways that they can use it to help in their clinic when they’re reviewing patients, that they can now use it more often and they encourage the other clinicians, but I guess it kind of needed a clinician to know that." [Staff 8]* | *"The possible barrier is a lack of clear clinical leadership: Why are we doing this? Where are we trying to get to? What's our North Star? What's our vision for this service? How does this speak to the long-term plan? What's in it for us, as in clinicians? More importantly, more importantly, what's in it for our patients to use?” [Staff 14]* |
|  |  |  | **Communication** | Communications around service promotion and training facilitated clinician engagement, but others commented that the strategy was at times unrealistic and misguided. | *"...we had all this training and that [clinical lead] is very heavily involved. So [clinical lead] helps to promote its presence in the Department…" [Staff 6]* | *"newsletters and that kind of died off the more we didn’t back. So we were putting a lot of effort into it and then we just, kind of, got the impression that they just weren’t going to be engaged at all. So we took a step back a bit from it because it was just a lot of work was going into trying to get the engagement levels higher and it just wasn’t paying off." [Staff 8]* |
|  |  |  | **Evaluation** | Lack of evaluation activities. | *N/A* | “I think we’ve not really been asked for feedback, I guess, that’s what you’re doing today. So that’s probably the limitation, there’s no PDSA as far as I’ve been aware. I’ve not been showcased any PDSA work, it’s just been done" [Staff 13] |
|  |  | **Organisational support** | | Lack of tangible organisational support. | *N/A* | *"I think there’s been a lot of generally supportive noises and feelings that this is a good thing to be doing, but it’s very much left up to busy clinicians to make sure that it continues and I’m not sure that that’s really viable long-term." [Staff 5]* |
|  |  | **Clinician factors** | **Attitude towards innovation** | RM service not seen as a priority in busy, time-pressured clinics. | *N/A* | *"I think just up take in clinic might take a bit longer because there isn’t a strong … I don’t feel there’s a strong benefit versus the time it takes. I don’t think it’s equal, and I definitely don’t think it’s beneficial yet there in that context" [Staff 13]* |
|  |  | **Barriers at roll-out sites** | **Leadership – communication and champions** | Poor leadership engagement, for example lack of a regular ‘in-person’ presence and effective champions. | *N/A* | *"…the show and tell was only held at Guy’s and St. Thomas's and was only attended by Guy's and St. Thomas's clinicians, so it would be reasonable to say that Lewisham and King's clinicians could be well excused for not knowing a great deal about the service."* *[Staff 14]* |
|  |  |  | **Organisational characteristics** | A central approach to patient management at roll-out sites was challenging. Plus, potential lack of appreciation of contextual differences. | *N/A* | *"But they give very valid reasons to why this may not be the one size that fits all. Everybody practices in very different ways" [Staff 11]* |
|  |  |  | **Clinician factors – attitude towards innovation** | Distrust and feeling like unequal partners. | *N/A* | *"There was always amongst clinician’s certain distrust about anyone who doesn’t work in your own trust. You don’t want your data somewhere else; you don’t want someone who isn’t your own nurse or your own doctor looking after patients. Someone else gives you an assessment, you always distrust, and you bring them over and you examine them yourselves, or you’re sent their scans over and you’ll get it relooked at by your own radiologist." [Staff 2]* |
|  | **Outer context** | **Inter-organisational environment** | | Difficulties surrounding patient management arising from a centralised approach based at the pilot site. | *N/A* | *"… she [DPC] has had episodes where she’s to try to contact clinicians at other sites who’s patients RAID scores have gone up and not gotten any response" [Staff 2]* |
|  |  | **Climate** | | The coronavirus pandemic reduced clinicians’ capacity to implement the RM service, through reduced manpower, disruption to clinical services and changing patient needs. | *N/A* | *“I think during the pandemic, because we lost half of our nurses, or more than half our nurses to critical care, we didn't have the capacity to really manage it as effectively as we would have done" [Staff 4]* |
|  | | | | | **Trust-wide level** | **Scale-up** |
| **Sustainment** | **Feasibility** | | | Wider scale up could be feasible, especially in smaller trusts. | *N/A* | *"I think that the system that’s in place now will be really well received especially in smaller trusts, smaller departments, if this is rheumatology only." [Staff 13]* |
|  | **Innovation factors** | **Innovation characteristics** | **Digital platform** | Ensuring the system has inbuilt flexibility to respond to local and evolving needs, and addressing current unmet needs, in particular the lack of integration with other key platforms. | *“So in five years the system will have to integrated with the electronic record, it cannot be a parallel system if it’s going to be sustainable.” [Staff 10]* | *"but how it works for us won’t be how it works for, for example, Lewisham or Kings because they have very different patient cohorts and very different challenges on their clinics I imagine. So it won’t be a one type fits every hospital. It will have to be adapted" [Staff 6]* |
|  |  |  | **Administrative support** | Administrative support should be increased in line with service expansion, feature at all relevant clinical sites, and include properly trained staff, information technology support and improved infrastructure. | *"We can’t just have one digital pathway coordinator dealing with all these messages coming through because that’s not safe for a service either. So we need enough people who are able to manage this workload and we have to be realistic that it is other channel for patients to get in contact with us and that we need to think about how we support that the best way. " [Staff 8]* | *N/A* |
|  |  |  | **Service design** | Improving patient guidance to promote self-management, to ensure patient safety and help manage workloads. | *"we have to be really careful that we make it a, sort of, patient initiated follow-up, rather than we are doing everything for those patients. That they are encouraged to go the right way to do something for their health and that if they’re flaring rather than just text, you know, waiting for their monthly PROM to come through to say that they’re flaring is that they actually contact us and say I’m flaring. I need support." [Staff 8]* | *N/A* |
|  |  | **Fit to clinicians** | | Important to prioritise clinician needs alongside patient needs. | *"it has to be done with the clinician also in mind and not just the patient" [Staff 11]* | *N/A* |
|  | **Inner context** | **Leadership** | **Leadership characteristics** | Strong leadership with good listening skills needed. | *"… So I think strong leadership, strong communication, really good listening, understanding what the nuanced challenges of a local problem might be, and lack of resource, so having the resources to manage the system" [staff 14]* | *N/A* |
|  |  |  | **Communication** | Effective communications strategy that includes regular stakeholder engagement and face-to-face time. | *"Continuing promotion of it, but I think once it becomes part of our everyday practice perhaps we wouldn’t need that. It’s about how we get to it being everyday practice, business as usual, rather than some new technology" [Staff 6]* | *"not to do it as a one off but have it as a recurring strategy." [Staff 10]* |
|  |  |  | **Evaluation** | Requirement for comprehensive and transparent evaluation activities. | *"I think we need to assess, actually, has it changed anything that we have done. Does it change any of our decision making? […] It would be nice to have examples of where it’s helped, and also examples from patients where it hasn’t helped...” [Staff 11]* | *"...there needs to be a very good report of the experience here, there’d need to be very clear advice on the resources needed and the potential gains that can be made by doing it…" [Staff 12]* |
|  |  | **Organisational support** | | Need for robust organisational support. | “*Trust Board and the Senior Team they’re incredibly excited about it […] but it doesn’t then really translate into anything. … there isn’t much support in terms of, you know, well let’s see how we can make this sustainable…" [Staff 5]* | *"We had our own app, when I first joined this trust, and we couldn't afford to keep it. So I think if this required us to pay, we wouldn't be able to afford to keep it.” [Staff 16]* |
|  |  | **Clinician factors** | **Readiness for change** | Need for culture change and consideration of readiness for change. | *“…it's around culture change. And I think it's around thinking differently and doing things differently.” [Staff 14]* | *"How ready are the other sites to adopt the service? How ready are they to adopt that way of working?” [Staff 14]* |

# **Supplementary Data S4. Lay summary**

Rheumatoid arthritis is a chronic, fluctuating, joint disease. Patient Reported Outcome Measures (PROMs), which are reports from patients about how they feel or function, have been shown to support patient-centred care. Remotely monitoring PROMs between clinic visits has the potential of improving patient care and care planning. In this study, we evaluated a remote monitoring (RM) service that collected PROMs and text messages from patients with RA attending outpatient clinics across six National Health Service (NHS) hospitals. We gathered data by several means, including conducting an online patient survey and interviews with patients and staff. Patients were involved throughout the study, to ensure that our findings were meaningful and relevant to patients. Patients were overwhelmingly positive about the RM service, whereas staff views were more mixed. Results suggest that equal levels of patients and staff engagement with the service are important for sustainment going forward. Patients and staff generally felt the RM service could lead to improved patient-centred care and clinical management. These findings further our understanding of the challenges of running RM services for patients with RA in routine care settings, across multiple hospitals. In addition, our findings may provide useful learning for others looking to provide similar RM services for patients with other chronic conditions.
